# Comprehensive miRNA sequence analysis reveals survival differences in diffuse large B-cell lymphoma patients

**DOI:** 10.1186/s13059-014-0568-y

**Published:** 2015-01-29

**Authors:** Emilia L Lim, Diane L Trinh, David W Scott, Andy Chu, Martin Krzywinski, Yongjun Zhao, A Gordon Robertson, Andrew J Mungall, Jacqueline Schein, Merrill Boyle, Anja Mottok, Daisuke Ennishi, Nathalie A Johnson, Christian Steidl, Joseph M Connors, Ryan D Morin, Randy D Gascoyne, Marco A Marra

**Affiliations:** Canada’s Michael Smith Genome Sciences Centre, British Columbia Cancer Agency, 675 West 10th Avenue, Vancouver, BC V5Z 1 L3 Canada; Department of Medical Genetics, University of British Columbia, Vancouver, Canada; Centre for Lymphoid Cancer, Department of Experimental Therapeutics, British Columbia Cancer Agency, 675 West 10th Avenue, Vancouver, BC V5Z 1L3 Canada; Department of Molecular Biology and Biochemistry, Simon Fraser University, Burnaby, British Columbia Canada; Department of Pathology and Laboratory Medicine, University of British Columbia, Vancouver, Canada

## Abstract

**Background:**

Diffuse large B-cell lymphoma (DLBCL) is an aggressive disease, with 30% to 40% of patients failing to be cured with available primary therapy. microRNAs (miRNAs) are RNA molecules that attenuate expression of their mRNA targets. To characterize the DLBCL miRNome, we sequenced miRNAs from 92 DLBCL and 15 benign centroblast fresh frozen samples and from 140 DLBCL formalin-fixed, paraffin-embedded tissue samples for validation.

**Results:**

We identify known and candidate novel miRNAs, 25 of which are associated with survival independently of cell-of-origin and International Prognostic Index scores, which are established indicators of outcome. Of these 25 miRNAs, six miRNAs are significantly associated with survival in our validation cohort. Abundant expression of miR-28-5p, miR-214-5p, miR-339-3p, and miR-5586-5p is associated with superior outcome, while abundant expression of miR-324-5p and NOVELM00203M is associated with inferior outcome. Comparison of DLBCL miRNA-seq expression profiles with those from other cancer types identifies miRNAs that were more abundant in B-cell contexts. Unsupervised clustering of miRNAs identifies two clusters of patients that have distinct differences in their outcomes. Our integrative miRNA and mRNA expression analyses reveal that miRNAs increased in abundance in DLBCL appear to regulate the expression of genes involved in metabolism, cell cycle, and protein modification. Additionally, these miRNAs, including one candidate novel miRNA, miR-10393-3p, appear to target chromatin modification genes that are frequent targets of somatic mutation in non-Hodgkin lymphomas.

**Conclusions:**

Our comprehensive sequence analysis of the DLBCL miRNome identifies candidate novel miRNAs and miRNAs associated with survival, reinforces results from previous mutational analyses, and reveals regulatory networks of significance for lymphomagenesis.

**Electronic supplementary material:**

The online version of this article (doi:10.1186/s13059-014-0568-y) contains supplementary material, which is available to authorized users.

## Background

Diffuse large B-cell lymphoma (DLBCL) is an aggressive form of non-Hodgkin lymphoma (NHL) that accounts for 30% to 40% of newly diagnosed lymphomas. Molecular profiling has revealed that the activated B-cell-like (ABC) and germinal center B-cell-like (GCB) subtypes of DLBCL are defined by their derivation from different cells of origin and exhibit differential response to chemotherapy [1]. In particular, the current combination of cyclophosphamide, doxorubicin, vincristine, prednisone, and rituximab chemotherapy (R-CHOP) yields inferior outcomes in patients with the ABC subtype compared to patients with the GCB subtype [1]. Thus, these subtype assignments add prognostic value to the widely used International Prognostic Index (IPI) that constitutes the clinical gold standard for identifying patients with poor prognosis [2,3]. Although gene expression signatures and single gene mutation (or expression)-based prognosticators have been described [4], many of these molecular features are surrogates for either the IPI or cell-of-origin (COO) subgroups. As such, the identification of additional biomarkers and therapeutic targets may offer the possibility of improved tools for clinical management of NHL.

microRNAs (miRNAs) are 17–25 nucleotide RNA molecules that regulate gene expression at the post-transcriptional level. Mature miRNAs predominantly act by directing the miRISC complex to complementary miRNA binding sites located on messenger RNAs (mRNAs), which results in cleavage or translational repression of these mRNA targets [5]. Many miRNA signatures have been identified in cancers [5], and several miRNAs, including miR-155 and the miR-17-92 cluster, have expression patterns that distinguish DLBCL from non-malignant B-cells [6]. Expression of several miRNAs, including miR-155, miR-21, and miR-221, differ between the ABC and GCB DLBCL subtypes [7]. In addition, miR-21 expression in tumor cells [7] and serum [8] has been shown to be associated with DLBCL patient prognosis. Subsequent to this finding, several groups [9-12] performed survival analyses on larger DLBCL patient cohorts using qPCR-based strategies or miRNome-wide microarrays and identified miRNAs that were associated with survival, including miR-21, miR-222, miR-23a, and miR-27a.

Deep sequencing of miRNA (miRNA-seq) provides a unique opportunity to catalog the repertoire of miRNA expression and study miRNA dysregulation comprehensively. miRNA-seq has been used to discover candidate novel miRNAs at various stages of B-cell development [13] and in NHL cell lines [14]. However, as far as we are aware, miRNA-seq has not yet been used to profile DLBCL patient samples.

Here we report on the miRNA-seq expression profiles of 92 DLBCL tumors and 15 purified benign centroblast fresh frozen samples, along with an integrated analysis of the DLBCL miRNome including clinical annotation, mutational and mRNA expression data. We also sequenced an additional 140 independent DLBCL formalin-fixed, paraffin-embedded tissue **(**FFPET) samples to validate our survival analyses. We identified candidate novel and known miRNAs expressed in DLBCL, including 25 miRNAs that appeared to be associated with survival independently of the established indicators of outcome (COO and IPI) in our Discovery Cohort. Of these 25, six miRNAs had their associations with survival replicated in our Validation Cohort. Abundant expression levels of miR-28-5p, miR-214-5p, miR-339-3p, and miR-5586-5p were associated with superior outcome, while abundant expression levels of miR-324-5p and NOVELM00203M were associated with poor outcome. Our comparisons of DLBCL miRNA expression to miRNA expression obtained from The Cancer Genome Atlas (TCGA) revealed that the miRNAs that are characteristic of DLBCL tend to have B-cell specific functions. In addition, our integrative miRNA:mRNA expression analysis provides evidence of miRNA-mediated repression of chromatin modification genes that are frequently inactivated by somatic mutations, reinforcing the notion that inactivation of these genes is linked to malignant progression in NHL.

## Results

### miRNA sequencing of fresh frozen DLBCL tumor and centroblast samples

Unlike miRNA microarrays, miRNA-seq provides, at least in principle, the opportunity to globally determine the presence and abundance of essentially all miRNAs across the entire DLBCL miRNome. To quantify expressed miRNAs, we sequenced 92 tumors from DLBCL patients (30 ABC-DLBCL, 41 GCB-DLBCL, and 21 unclassified-DLBCL; all of whom were treated with multi-agent chemotherapy (83 R-CHOP; 17 other regimens; for clinical characteristics see Additional file 1: Table S1 and Additional file 2: Table S2) and 15 purified benign centroblast samples. Each miRNA-seq library was sequenced to an average depth of 5.34 (range: 1.34-16.91) million reads, which we have found is generally sufficient to identify moderate-to-low-abundance miRNAs including those exhibiting modest expression differences between samples that may not be detected by hybridization-based methods [15].

**Figure 1:**
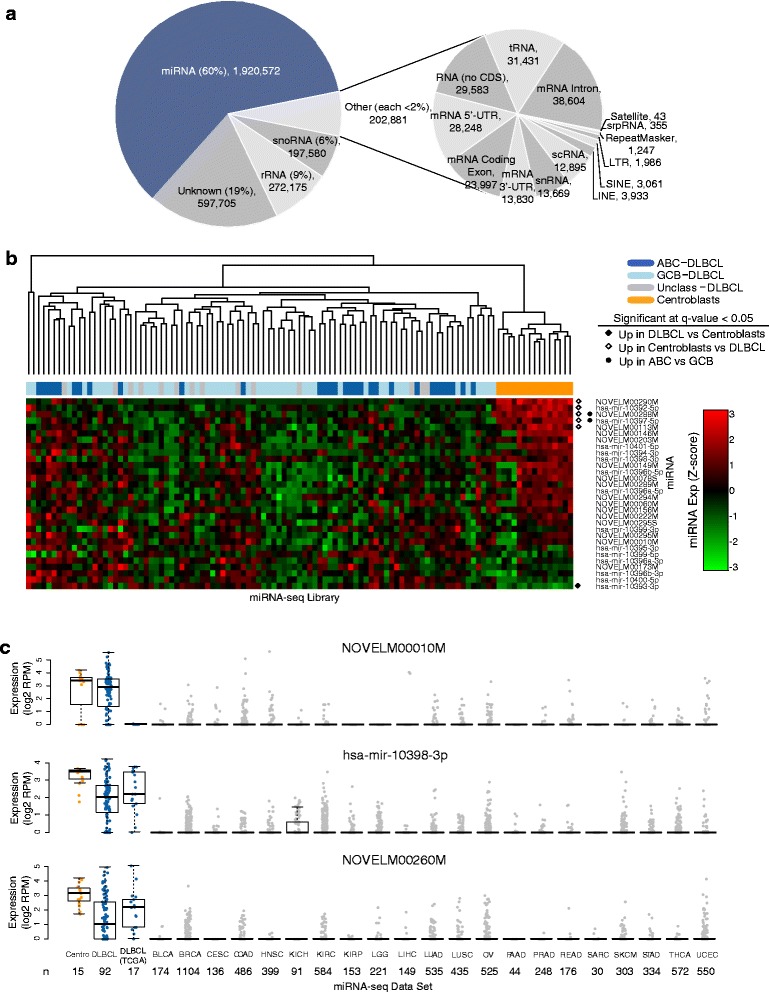
**Profiling miRNA in DLBCL. (a)** miRNA sequence analysis identifies several small RNA species, with the majority of reads aligning to miRNA loci. The pie chart depicts the proportion and origin of miRNA-seq aligned reads. Reported proportions are averaged across the 92 DLBCL and 15 centroblast libraries. **(b)** Expression of candidate novel miRNA across DLBCL and centroblast libraries. Column labels represent the type of sample: Dark Blue: ABC-DLBCL; Light Blue: GCB-DLBCL; Gray: Unclassified-DLBCL; Orange: Centroblasts. Row labels are annotated to indicate whether the miRNA was more abundantly expressed in a sample category. **(c)** Expression of B-cell enriched candidate novel miRNAs (NOVELM00010M, miR-10398-3p and NOVELM00260M) in DLBCL, centroblasts, and other cancers. BLCA: bladder urothelial carcinoma; BRCA: breast invasive carcinoma; CESC: cervical squamous cell carcinoma and endocervical adenocarcinoma; COAD: colon adenocarcinoma; HNSC: head and neck squamous cell carcinoma; KICH: kidney chromophobe; KIRC: kidney renal clear cell carcinoma; KIRP: kidney renal papillary cell carcinoma; LGG: brain lower grade glioma; LIHC: liver hepatocellular carcinoma; LUAD: lung adenocarcinoma; LUSC: lung squamous cell carcinoma; OV: ovarian serous cystadenocarcinoma; PAAD: pancreatic adenocarcinoma; PRAD: prostate adenocarcinoma; READ: rectum adenocarcinoma; SARC: sarcoma; SKCM: skin cutaneous melanoma; STAD: stomach adenocarcinoma; THCA: thyroid carcinoma; UCEC: uterine corpus endometrial carcinoma. Blue: DLBCL; Orange: Centroblast.

We observed that 310 known miRNAs (3p or 5p strands of 221 miRNA species in miRBase version 19) were expressed at levels of at least 10 reads per million (RPM) in at least 10% of the samples. Our threshold for calling expressed miRNAs (>10 RPM in >10% samples) was based on miRBase criteria [16] for high confidence miRNAs. In addition to miRNAs, which accounted for 60% of the aligned miRNA-seq reads, our pipeline also identified the expression of other classes of small RNAs. For example, an average of 9% of the aligned reads mapped to rRNAs and 6% to snoRNAs. Other non-coding RNAs (tRNAs, snRNAs, scRNAs) and DNA repeat elements were represented by fewer reads (Figure 1a; Additional file 3: Table S3).

### Novel miRNA discovery

We interrogated our 92 DLBCL miRNA-seq libraries to identify candidate novel miRNA species that were dysregulated in NHL. After sequence filtering, we enumerated 234 candidate novel miRNAs (that is, not identified in miRBase v19; Additional file 4: Table S4). The mean expression levels of these candidate novel miRNAs (average: 3.84 RPM; range: 0.00-4,979.00 RPM) were lower than that of the annotated miRNAs (average: 218.50 RPM; range: 0.00-131,200.00 RPM). Thirty of these putative miRNAs were expressed at levels of at least 10 RPM in more than 10% of DLBCL and centroblast samples, and this subset was used in subsequent analyses. Of these, five were more abundant in benign centroblasts than in patient samples, while one, miR-10393-3p, was more abundant in DLBCL patient samples than in centroblasts (Wilcoxon test BH q-value <0.05; log_2_ fold change >2). Two miRNAs (miR-10397-5p, NOVELM00288M) were more abundant in ABC-DLBCL (Wilcoxon test BH q-value <0.05; Figure 1c). This differential abundance indicated that expression of these candidate novel miRNAs might reveal regulatory pathways deployed in these DLBCL subtypes and therefore might be useful in the classification of tumors. To broadly survey the expression of these miRNAs in cancers, we analyzed their expression in 7,266 TCGA miRNA-seq samples from 21 other cancer types. Three miRNAs (NOVELM00260M, NOVELM00010M, and miR-10398-3p) were significantly more abundant (Wilcoxon test BH q-value <0.05; median of expression of miRNA in other cancers = 0) in B-cell contexts (DLBCL and centroblast samples; Figure 1b), suggesting that they may have functions enriched in, or specific to, B-cells. These 30 highly expressed candidate novel miRNAs were subjected to further analyses, in which our survival analysis revealed the associations with survival of some of them, while our integrative expression analysis revealed the potential lymphomagenic roles of others.

### miRNA expression in DLBCL

**Figure 2:**
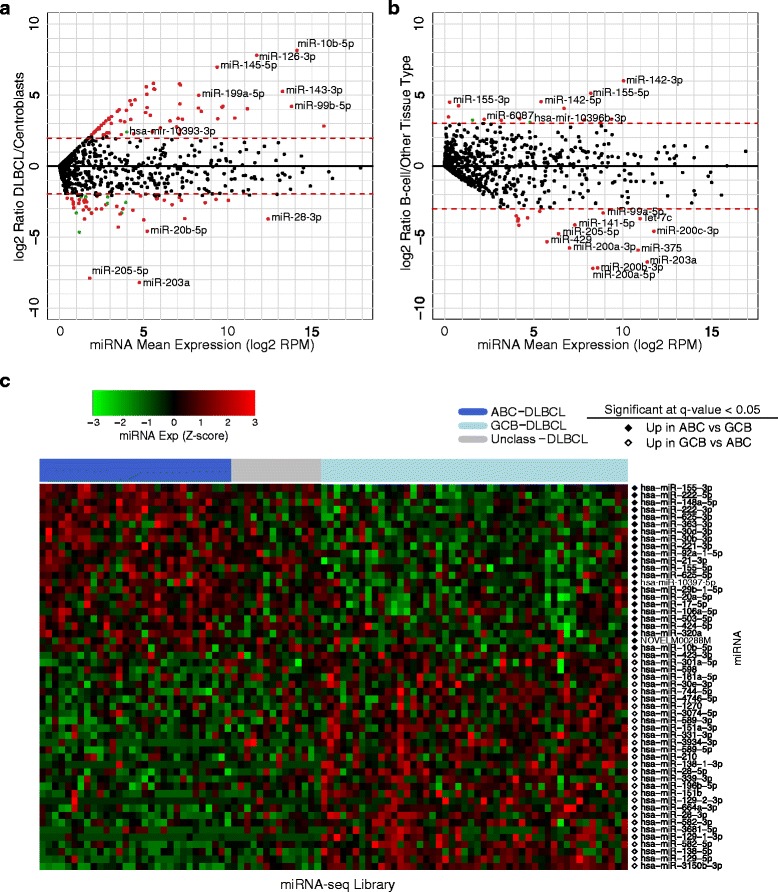
**Differential expression analysis reveals novel and known miRNAs.** Differential expression for each miRNA was calculated using the Wilcoxon ranked-sum test, and *P* values were multiple-test corrected using the BH algorithm. **(a)** MA (Log ratio (M) versus mean average (A) expression) plot showing differentially expressed miRNAs comparing DLBCL to centroblasts. **(b)** MA plot showing miRNA that are differentially expressed between the B-cell data sets (DLBCL and centroblasts) and all other TCGA cancer data sets. In both MA plots, significantly differentially expressed known miRNAs are represented by red dots, while significantly differentially expressed candidate novel miRNAs are represented by green dots. **(c)** Heatmap of differentially expressed miRNAs between the ABC and GCB DLBCL subtypes. Column labels represent the type of sample: Dark Blue: ABC-DLBCL; Light Blue: GCB-DLBCL; Gray: Unclassified-DLBCL. Row labels indicate if the miRNA is more abundant in a particular category of samples.

To obtain a comprehensive list of candidate novel and known miRNAs that are characteristic of DLBCL, we compared the expression of each miRNA in DLBCL samples with those of benign centroblasts obtained from our miRNA-seq data. We noted that 63 miRNAs exhibited increased abundance in DLBCL, while 39 miRNAs exhibited decreased abundance in DLBCL (Wilcoxon test BH q-value <0.05; log_2_ fold change > 2; Figure 2a). Of the miRNAs with increased abundance in DLBCL, only miR-125b-5p [17] and miR-34-5p [18] have previously been implicated in lymphomagenesis in mouse models.

To identify miRNAs that were more abundant in either ABC or GCB DLBCL subtypes, we performed differential expression analysis for each miRNA by comparing expression values between the two groups. Twenty-three miRNAs were more abundant in ABC-DLBCL, while 30 miRNAs were increased in abundance in GCB-DLBCL (Wilcoxon test BH q-value <0.05; Figure 2c). In addition, our analysis revealed that the miRNAs whose expression is increased in GCB-DLBCL appear to target transcripts that are known to be dysregulated in the formation of germinal center lymphomas [19]. These miRNA:mRNA pairs, which had anti-correlated expression in our data, include miR-181-5p:*BCL2*, miR-181a-5p/miR-28-5p/miR-3150-3p/miR-589-5p:*IFNAR1* and miR-129-5p/miR-3150b-3p/miR-28-3p:*IRF4* (Additional file 5: Table S5).

We further assessed expression levels of each candidate novel miRNA in a published HITS-CLIP data set obtained from primary effusion lymphoma cells (Haecker *et al*. [20]). We detected the expression of 12 of the candidate novel miRNAs in this external independent data set (≥1 RPM; ≥1 sample) (Additional file 6: Table S6), thus providing evidence that these 12 miRNAs do indeed interact with the Ago protein (a subunit of the RISC complex), and are bona-fide miRNAs. Further, in order to detect the expression of these candidate novel miRNAs using an orthogonal technology, we performed RT-qPCR on tumor samples. We tested four of the 12 miRNAs that were verified by HITS-CLIP (NOVELM00060M, NOVELM00113M, NOVELM00222M, NOVELM00290M). These experiments confirmed the presence of all four of the tested miRNA (Additional file 7: Table S7).

### B-cell-enriched miRNA expression profiles

Given that miRNA expression is often cell-lineage-specific [21], we sought to identify B-cell-enriched profiles using a pan-cancer miRNA-seq analysis. We compared our B-cell data set (DLBCL and centroblast samples) to TCGA data from 21 other cancer types to identify miRNAs that were differentially expressed between our B-cell data set and all other TCGA cancer types. The 17 DLBCL cases from the TCGA data set were included in the B-cell test group for these comparisons. This analysis identified 15 miRNAs that were significantly more abundant in B-cell contexts when compared with each of the 21 cancer types (Wilcoxon test BH q-value <0.05; log_2_ fold change >3; Figure 2b; Additional file 8: Table S8). miR-142-3p was the most significantly increased, displaying a 64-fold increase in B-cell contexts (Additional file 9: Figure S1). Interestingly, miR-142 expression was also more abundant in the benign centroblast samples when compared with the DLBCL patient samples, suggesting that miR-142 could play an important role in normal B-cell function. Of the miRNAs that were significantly more abundant in B-cell contexts when compared with other cancers, abundant expression of miR-3150b-3p, miR-6087, and miR-4491 in B-cells has not been previously reported. Our analysis indicated that miR-4491 may be involved in suppressing the expression of genes associated with the innate immune response (GO:0045087) (Additional file 5: Tables S5 and Additional file 10: Table S9). Supporting this notion is the observation that several of these immune response genes are also frequently less abundantly expressed in GCB-DLBCL, including *IFNAR1*, *TLL2*, *TLR4*, and *TLR8* [19].

We found that 17 miRNAs were significantly decreased in abundance in our B-cell data set when compared to other cancers (Wilcoxon test BH q-value <0.05; log_2_ fold change < −3; Figure 2b). Of note, members of the miR-200 family (miR-200a-3p, miR-200a-5p, miR-200b-3p, miR-200b-5p, miR-200c-3p, and miR-200c-5p) were the most significantly decreased in abundance. In agreement with this, it has been reported that reduced expression of miR-200 family members results in more aggressive DLBCL through the de-repression of *ZEB1* [22].

### Integrative analysis of miRNA and mRNA expression

**Figure 3:**
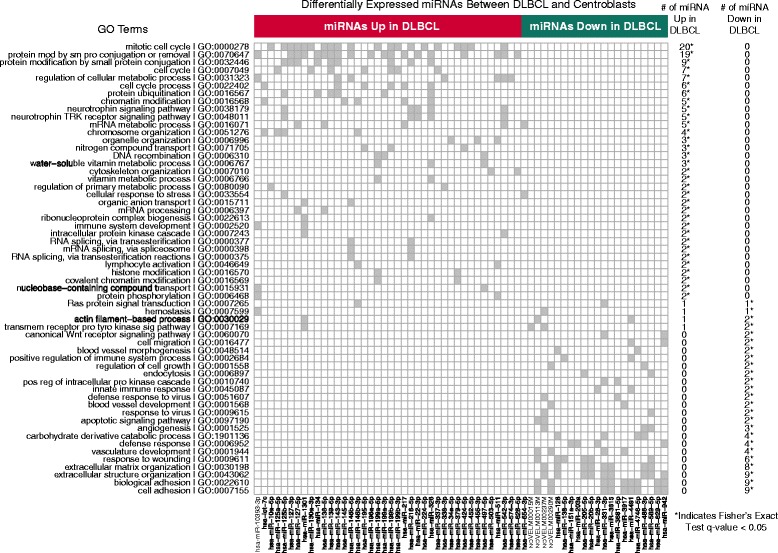
**Candidate miRNA:mRNA interactions in DLBCL.** Chart showing Gene Ontology (GO) Biological Process terms that were enriched for genes involved in reciprocally expressed miRNA:mRNA pairs. Each column indicates a miRNA that is either increased or decreased in abundance in comparisons between DLBCL and centroblasts. Each row represents a GO term. A grey bar indicates that the GO term is significantly enriched in the candidate gene targets of one or more miRNA. Only GO Terms that have been enriched by targets of at least two miRNAs are shown. The number of miRNAs in each category are shown on the right.

miRNA expression can regulate translation and mRNA stability. Considering the latter mechanism, we assessed the relationship between aberrantly expressed miRNA and mRNA abundance. Using the miRNA and mRNA profiles from the 92 DLBCL and 15 centroblast samples, we identified putative miRNA:mRNA regulatory interactions (Additional file 9: Figure S2; Additional file 5: Table S5). miRNAs that were more abundantly expressed in DLBCL appeared to interact with genes enriched in the Gene Ontology (GO) biological processes related to cell cycle, metabolic processes, chromatin modification, protein modification, nerve growth factor signaling pathways, and organelle organization (Figure 3, Additional file 10: Table S9). Conversely, miRNAs that were expressed at lower levels in DLBCL appeared to interact with genes that were enriched in GO biological processes related to extracellular organization, cellular adhesion, defense and wounding responses, actin cytoskeleton organization, blood vessel morphogenesis, and endocytosis (Figure 3, Additional file 10: Table S9).

**Figure 4:**
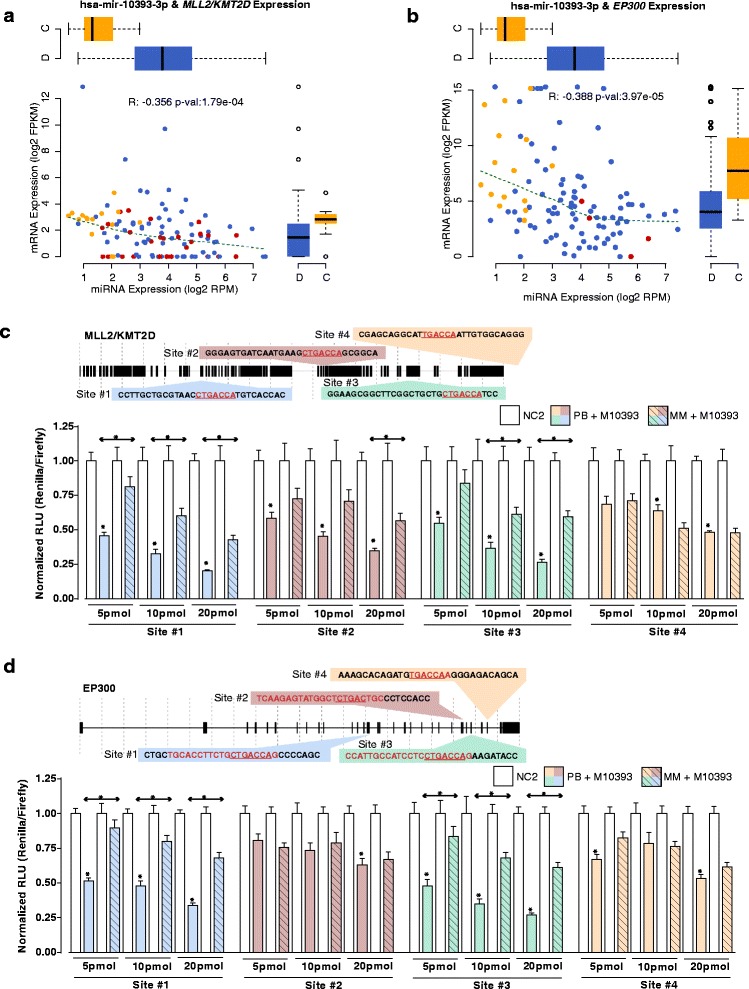
**Chromatin modifying genes may be targets of miRNA-mediated expression in DLBCL.** miR-10393-3p is involved in miRNA:mRNA interactions with chromatin modifiers *MLL2/KMT2D* and *EP300*. **(a, b)** miRNA and mRNA display anti-correlated expression patterns and the mRNA has a predicted binding site for miR-10393-3p (M10393). Orange dots represent centroblast libraries, red dots represent DLBCL libraries with a somatic mutation in *MLL2/KMT2D* or *EP300*, respectively, and blue dots represent DLBCL patient samples without the mutation. The boxplots to the top and right of each scatter plot summarize miRNA and mRNA expression in DLBCL (‘D’) and Centroblasts (‘C’); **(c, d)** Top: Schematic representations of the putative miR-10393-3p binding sites on *MLL2/KMT2D* or *EP300*. Putative seed regions within each site are underlined and in red font. Bottom: Dose response of miR-10393-3p miRNA activity in HEK-293 cells was assessed using a psiCHECK2 dual luciferase reporter construct containing each of the putative *MLL2/KMT2D* or *EP300* binding sites. Activity is measured as Renilla luciferase normalized to Firefly luciferase to control for transfection efficiencies. The data were shown as normalized relative luciferase units (RLU) with respect to the corresponding dose of the control mimic and are representative of three independent experiments (mean ± SEM). Statistically significant comparisons between the co-transfected M10393 miRNA and the NC2 control for the perfect binding reporter vector are noted over the solid colored bars. Statistically significant comparisons between perfect binding and mismatch constructs are indicated above double-headed arrows. *P <0.05. White bars, NC2 negative control mimics; Solid colored bars, M10393 mimics on perfect binding (PB) sites; Striped colored bars, M10393 mimics on mismatched (MM) sites.

miR-10393-3p, the candidate novel miRNA that was more abundantly expressed in DLBCL than in centroblasts, appeared to interact with transcripts from chromatin modifier genes. These genes included *BRPF3*, *RCOR1*, *WHSC1L1*, *WHSC1*, *CHD6*, *KDM5C*, *SMARCA4*, *MLL2/KMT2D*, and *EP300*. Although the number of targeted chromatin modifiers was not sufficient to statistically enrich the chromatin modification GO Term (GO:0016568), two of these candidate targets (*MLL2/KMT2D* and *EP300*) are frequently mutated in NHL [23] (Figure 4a and b). This result is compatible with the notion that chromatin modification may be dysregulated in NHL patient samples by both miRNA-mediated repression and by somatic mutation. These two interactions were further validated by luciferase assays, where over-expression of miR-10393-3p inhibited the luciferase activity of constructs containing each of the four predicted *MLL2/KMT2D* binding sites (Figure 4c). Sites 1 to 3 of *MLL2/KMT2D* contain the full putative miR-10393-3p binding site whereas site 4 contains a 1 bp mismatch. The mismatch in site 4 may explain the reduced sensitivity to overexpression of miR-10393-3p for both the perfect binding and mismatched constructs. The effect of miR-10393-3p over-expression was similar for each of the four predicted *EP300* binding sites (Figure 4d), where sites 1 and 3 of *EP300*, which contain the putative miR-10393-3p binding site, were more sensitive to miR-10393-3p overexpression than sites 2 and 4, which contain a 2 bp and 1 bp mismatch, respectively.

### miRNAs associated with DLBCL patient outcome

#### R-CHOP-treated Discovery Cohort

Given that approximately 40% of DLBCL patients succumb to their disease, and that prognostic markers for improved risk stratification are needed, we sought to identify miRNAs which are associated with patient survival. For our survival analyses, we considered the subset of the 92 patients that were uniformly treated with R-CHOP (n = 83; 29 ABC-DLBCL, 41 GCB-DLBCL, and 13 unclassified-DLBCL). This cohort is hereafter referred to as the ‘Discovery Cohort’. The characteristics of our study population, including the parameters that comprise the International Prognostic Index (IPI), are shown in Additional file 1: Table S1. Originally proposed in 1993 [2], the IPI is based on treatment with CHOP, and its modernized version, the R-IPI [3], which reflects the changes resulting from addition of rituximab to the original CHOP regimen, remain the primary clinical tools used to predict outcome for patients with DLBCL [3]. However, even though both IPI and COO segregated patients into low and high clinical risk groups in our data set, the log rank *P* values were not significant (*P* value >0.05; Additional file 9: Figure S3).

#### miRNAs associated with patient survival

**Figure 5:**
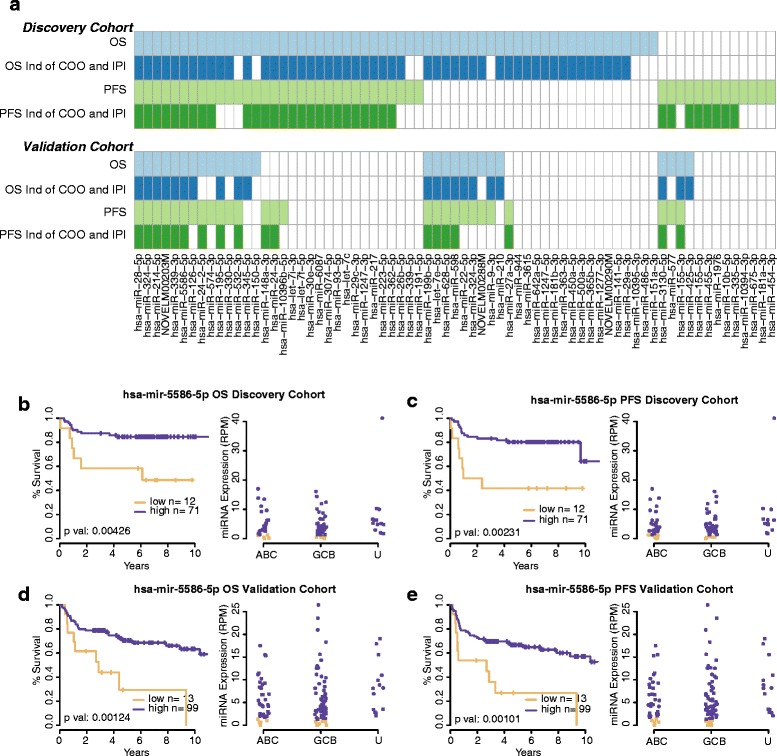
**Survival analyses in the Discovery and Validation Cohorts. (a)** miRNAs that were associated with OS and/or PFS in the Discovery Cohort (n = 83), and Validation Cohort (n = 112). Six miRNAs (miR-28-5p, miR-324-5p, miR-214-5p, miR-339-3p, miR-5586-5p, NOVELM00203M) were found to be associated with OS and PFS, independently of COO and IPI, in both the Discovery and Validation Cohorts. Light Blue: miRNA associated with OS; Dark Blue: miRNA associated with OS independently of COO and IPI; Light Green: miRNA associated with PFS; Dark Green: miRNA associated with PFS independently of COO and IPI; miR-5586-5p Kaplan-Meier curves and scatter plots of expression in (ABC)-DLBCL, (GCB)-DLBCL, and (U)nclassified-DLBCL: **(b)** Discovery Cohort OS, **(c)** Discovery Cohort PFS, **(d)** Validation Cohort OS, **(e)** Validation Cohort PFS. Plots for the other five validated miRNAs are shown in Additional file 9: Figure S7.

To identify miRNAs with expression patterns associated with patient overall survival (OS) and progression-free survival (PFS), we performed log-rank tests on X-tile-derived [24] low and high expression patient groups of each miRNA. This revealed that 58 and 45 miRNAs are associated with OS and PFS, respectively (log-rank q-value <0.05). Seven of these miRNAs have previously been associated with DLBCL patient survival: miR-330 [9], miR-93 [10], miR-148a [10], miR-155 [6], miR-151 [10], miR-181a [11], and miR-28 [10]. To determine which of these miRNAs were associated with OS and PFS independently of the two established indicators of DLBCL patient outcome (COO and IPI), we performed Cox proportional hazards (PH) multivariate analysis on the X-tile-derived low and high expression patient groups for each miRNA, along with COO and IPI patient status. The results of this analysis revealed that 25 miRNAs were associated with OS and PFS independently of COO and IPI (*P* value <0.05; Figure 5a; Additional file 11: Table S10).

#### R-CHOP-treated Validation Cohort

To measure the association of these miRNAs with OS and PFS, we performed miRNA-seq on the diagnostic FFPET biopsies of 140 DLBCL patients treated with R-CHOP. We utilized FFPET samples as these were readily available to us. This FFPET cohort included 28 cases that were also in the fresh frozen Discovery Cohort; the 112 unique cases represent an independent Validation Cohort. The characteristics of our validation study population are shown in Additional file 12: Table S11. We used the 28 common samples to explore the potential effects of formalin fixation. To do so, we compared miRNA expression from FFPET and fresh frozen samples of these 28 cases using hierarchical clustering. The result was two clusters: one consisting predominantly of fresh frozen samples, and the other consisting predominantly of FFPET samples (Additional file 9: Figure S6). This result indicates that FFPET samples are more similar to other FFPET samples than they are to matched fresh frozen samples from the same patient, and is in agreement with a previous study that reports on RNA degradation observed in FFPET miRNA-seq data [25].

#### Validation of miRNAs associated with patient survival

Despite the differences between fresh frozen and FFPET miRNA-seq expression profiles (Additional file 9: Figure S6), our survival analyses (as performed in the Discovery Cohort) based on expression profiles obtained from the Validation Cohort replicated several associations of miRNA expression with OS and/or PFS that had been identified in the Discovery Cohort. Specifically, we validated the association of 28 of 58 miRNAs (48%) with OS, and the association of 19 of 45 miRNAs (32%) with PFS (log-rank *P* value <0.05). Our analysis also validated the association of six of 25 miRNAs (24%) with both OS and PFS independent of COO and IPI (Cox PH *P* value <0.05; Figure 5; Additional file 11: Table S10). These six miRNAs include miR-28 which was previously associated with survival in DLBCL [10] and five other miRNAs that have not previously been associated with DLBCL patient survival. We observed that abundant expression levels of miR-28-5p, miR-214-5p, miR-339-3p, and miR-5586-5p are associated with superior outcome, while abundant expression levels of miR-324-5p and NOVELM00203M are associated with poor outcome (Figure 5a). Representative Kaplan-Meier curves and expression values for miR-5586-5p in both the Discovery and Validation Cohorts are displayed in Figure 5b-e, while results for the other five miRNAs are displayed in Additional file 9: Figure S7.

#### miRNA expression profiles associated with patient survival

**Figure 6:**
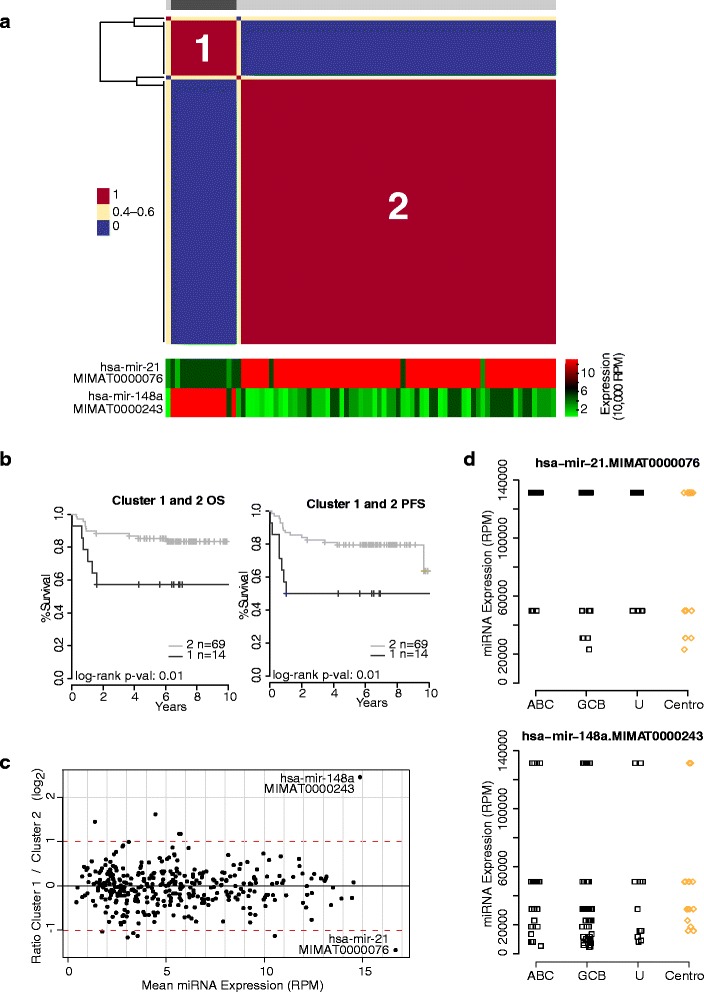
**NMF Identifies two clusters of DLBCL patients with distinct miRNA and outcome profiles.** We performed non-negative matrix factorization (NMF) clustering on 83 R-CHOP treated DLBCL patient samples, using the miRNA expression obtained from miRNA-seq data. **(a)** NMF yielded two clusters of patients (see Additional file 9: Figure S4) that had distinct differences in their outcomes. Patients in cluster 1 are indicated by dark gray bars, while patients in cluster 2 are indicated by light gray bars. Below the consensus matrix is a heatmap showing the expression of miR-148a and miR-21 in each patient. **(b)** Kaplan-Meier curves showing overall survival and progression-free survival of patients in both clusters. Patients in cluster 1 exhibit inferior outcome compared to those in cluster 2. **(c)** To identify which miRNAs were characteristic of each cluster, we identified the differentially expressed miRNA between the two clusters. The MA plot shows that miR-21 abundance is increased in cluster 2 patients, while miR-148a abundance is decreased in cluster 1 patients (Wilcoxon test q-value <0.05). **(d)** Expression patterns of miR-148a and miR-21 are discontinuous. miRNA expression in DLBCL patient samples is indicated with black squares, while expression in centroblast samples is indicated with orange diamonds.

We next sought to determine whether DLBCL patients could be stratified using their miRNA expression profiles. Unsupervised non-negative matrix factorization (NMF) consensus clustering (Additional file 9: Figure S4), using only the miRNA expression profiles of the 83 R-CHOP treated patients, identified an optimum of two groups of patients (Figure 6a) with distinct outcome correlations (Figure 6b) and miRNA expression patterns (Figure 6c). These two groups did not differ based on any clinical characteristics, including age, sex, LDH level, number of extranodal sites, cell-of-origin subtype, or other parameters such as presence of a chromosomal break-apart at *BCL2*, *BCL6*, or *MYC* (Chi-square test *P* value >0.05). However, two miRNAs were significantly differentially expressed between the groups. In the cluster of patients with poorer outcome, miR-148a was increased in abundance and miR-21 was decreased in abundance compared to the cluster of patients with superior outcome (Figure 6a).

Low expression of miR-21 in tumors [7] and in serum [8] of DLBCL patients has been associated with poor outcome, and high expression of miR-148a has been associated with poor survival in a COO-based classifier [10]. In our Discovery Cohort, miR-21 and miR-148a expression patterns were significantly associated with OS and PFS (Additional file 9: Figure S5a); and this trend is also evident in our Validation Cohort, although not at statistically significant levels (Additional file 9: Figure S5b). Both of these miRNAs appear to be highly expressed and highly variable in DLBCL and centroblast samples and exhibit discontinuous expression patterns (Figure 6d), suggesting that they may be robustly detected in clinical samples.

Our integrative analysis revealed that miR-148a candidate targets included genes associated with immune response (GO:0006955); for example, *AMICA1*, *CCR5*, *CD28*, *CD3G*, *CD8A*, *CD96*, *CLEC10A*, *CSF1*, *CTSW*, *CXCL12*, *CXCL16*, *GZMM*, *ITK*, *LCP2*, *MX2*, *NUB1*, *OASL*, *PRKCQ*, *SAMHD1*, *SELL*, *SIGIRR*, *TMEM173*, and *XCL1*. Of note, *CXCL12* is a chemokine receptor which plays a role in germinal center homing [26] and *CCR5* expression is associated with the transformation of mucosa-associated lymphoid tissue (MALT) lymphoma to DLBCL [27]. The observation that several immune response genes are targets of miR-148a is compatible with the notion that DLBCL patients with higher miR-148a expression levels exhibit attenuated immune responses due to the repression of immune response genes. Further, six of the genes (*CD28*, *CD3G*, *CD8A*, *ITL*, *LCP2*, *PRKCQ*) are part of the T-cell receptor pathway, suggesting that T-cell interactions could be disrupted in patients with poor prognosis.

## Discussion

We report here on the first deep sequencing analysis of the DLBCL miRNome. We profiled 92 patient samples (including samples from 83 uniformly R-CHOP treated patients) and 15 normal centroblast fresh frozen samples and analyzed the expression of known and candidate novel miRNAs. We further sequenced miRNAs from 140 FFPET-derived DLBCL samples as a Validation Cohort for our survival analyses. In addition, our integrative miRNA:mRNA expression analysis was used to inform on the potential impact of miRNA dysregulation on B-cell biology and on DLBCL pathogenesis. These data provide a genome-wide view of miRNA expression and dysregulation in DLBCL.

Existing miRNA profiling efforts in DLBCL patient cohorts have largely been probe-based [6,7,9,12,28], which are biased toward detection of known miRNAs at the expense of identification of candidate novel miRNAs. miRNA-seq does not have this same limitation, and thus provides an opportunity to identify candidate novel miRNA species. A previous miRNA-seq analysis of 3 DLBCL cell lines identified more than 200 novel miRNAs [14]. (Additional file 13: Table S12) Here we report on the discovery of an additional 234 novel miRNAs in 92 DLBCL tumor samples, where 30 of these were frequently expressed across DLBCL tumor samples and 29 were also detected (median RPM >1) in the FFPET Validation Cohort (n = 112) (Additional file 6: Table S6). Of note, miR-10393-3p appeared to be more abundant in DLBCL tumor samples than in benign centroblasts. Further, our analysis is compatible with the notion that miR-10393-3p may play a role in the pathogenesis of DLBCL through attenuation of chromatin modifier gene expression.

DLBCL tumors have been shown to have miRNA expression profiles distinct from those of benign B-cells, and dysregulated miRNAs have functional roles in B-cell differentiation and lymphomagenesis [13]. To shed light on the functions of dysregulated miRNAs, we performed an integrative miRNA and mRNA expression analysis which provided a transcriptome-wide view of miRNA:mRNA interactions that may be acting in DLBCL. This analysis indicated that the miRNAs that are abundantly expressed in DLBCL may modulate cell cycle regulation, cell metabolism and chromatin modification in disease progression. We and others [23,29] recently reported the frequent mutation of chromatin modification genes in NHL, illustrating the relevance of the epigenome in malignant progression. Our work here presents miRNA-mediated repression as another mechanism for the dysregulation of chromatin modification genes that are mutated in NHL. First, we show that the expression of a candidate novel miRNA (miR-10393-3p) is abundantly expressed in DLBCL when compared with centroblasts. Further, miR-10393-3p exhibits significant expression profiles that are anti-correlated with the expression profiles of 11 chromatin modification-related genes, including *MLL2/KMT2D* and *EP300,* which are recurrent targets of somatic mutation in NHL [23]. These results suggest DLBCL progression could proceed through mutations or miRNA-mediated repression as mechanisms that modulate the epigenome.

Given that DLBCL comprises molecularly distinct subtypes, we sought to identify differentially expressed miRNAs that were associated with these subtypes. miRNAs that were upregulated in ABC-DLBCL included members of the oncogenic miR-17-92 cluster (miR-106a, miR-17, miR-20a, miR-92a) [30], and others that have been implicated in lymphomagenesis in mouse models (miR-155 [17], miR-21 [31]). Although not previously implicated in the pathogenesis of ABC-DLBCL, miR-625 has been shown to regulate invasion and metastasis in gastric cancer by targeting and regulating the expression of ILK [32]. Members of the miR-29 family, including miR-29b, target the WNT signaling pathway by attenuating expression of DNMT3A and DNMT3B [33]. Members of the miR-30 family have been shown to bind to and regulate BCL6 in B-lymphocytes and lymphoma cells [34]. Thus, decreased expression of miR-30b in GCB-DLBCL could promote the germinal center phenotype through the de-repression of BCL6.

Previously, a pan-cancer miRNA analysis revealed that miRNA expression profiles tend to be tissue specific and can distinguish cancer samples of different cancer types from one another [21]. Another pan-cancer effort demonstrated that expression levels of miR-142 and miR-509 expression to be characteristic of lymphomas when compared with melanomas within a decision tree consisting of 25 cancer types [35]. Our comparison of DLBCL and centroblast miRNA expression data to similar data from TCGA cancers showed that the miRNAs that are frequently expressed in DLBCL (including 3 candidate novel miRNAs) tended to have B-cell enriched expression patterns and candidate functions and they are frequently dysregulated in B-cell lymphomas. For instance, miR-191 is part of a 6-miRNA signature that can discriminate B-lineage acute lymphoblastic leukemia (ALL) subgroups harboring specific molecular lesions [36]. miR-7 is abnormally increased in abundance in lymphoid cancers including childhood ALL [37] and follicular lymphoma [38]. miR-155 expression is known to be crucial in the B-cell germinal center transition through regulation of the master B-cell regulator *AID* [39], and its expression levels are crucial for normal B-cell function: over-expression of miR-155 is associated with DLBCL, while under-expression is associated with Burkitt lymphoma [40]. miR-142, the miRNA that displayed the most significant increase in abundance in B-cell contexts, has been shown to regulate B-cell stimulation by downregulating the expression of SAP, CD84 and IL-10 proteins [41]. miR-142 is also mutated in approximately 20% of DLBCL cases, where mutations in the seed region lead to a loss of binding activity to oncogenic *RAC1* and *ADCY9* mRNA transcripts and a possible gain of binding activity to transcriptional repressors *ZEB1* and *ZEB2* [42].

The ability to accurately predict response to therapy and survival is advantageous for DLBCL patient treatment planning. In this regard, there have been several efforts to explore the utility of miRNA expression. For example, Alencar *et al.* [11] investigated the prognostic value of 11 miRNAs using qPCR, while Montes-Monero *et al.* [10] similarly evaluated miRNA profiles in 36 patients using microarray-based technology. Our results reveal that the expression of 25 miRNAs is associated with both OS and PFS independently of established indicators of patient outcome (COO and IPI). We replicated our survival analyses in our FFPET-derived Validation Cohort. We utilized FFPET samples as these were available to us and fresh frozen samples were not. Studies have compared miRNA expression profiles obtained from FFPET and fresh frozen samples, and have shown that differences between profiles exist [25,43,44]. For example, miRNAs extracted from FFPET tend to have shorter average lengths [43], reduced purity [25], and higher expression levels than miRNAs from fresh frozen samples [44]. Despite the differences between our fresh frozen Discovery and FFPET Validation Cohorts, we replicated the robust association of six miRNAs (miR-28-5p, miR-214-5p, miR-339-3p, miR-5586-5p, miR-324-5p, NOVELM00203M) with OS and PFS independently of COO and IPI. The independent association of these miRNAs with OS and PFS suggests that there is heterogeneity within the groups derived from the COO and IPI classifications. Further, our integrative analysis indicated that the mRNA targets of NOVELM00203M are involved in cell adhesion (GO:0007155), reinforcing the importance of cell adhesion [45] in the pathogenesis of DLBCL. miR-28 has previously been associated with DLBCL patients outcome [10], and is a tumor-suppressor in Burkitt Lymphoma [46]. However, the other five miRNAs we identified as independent factors affecting survival of patients with DLBCL, miR-214-5p, miR-339-3p, miR-5586-5p, miR-324-5p, and NOVELM00203M have not previously been implicated in DLBCL outcome. Although beyond the scope of this study, these miRNAs may serve as the basis for a future prognostic tool and will inform further studies of DLBCL biology.

## Conclusions

We describe, for the first time, deep and comprehensive profiling of the DLBCL miRNome using miRNA-seq. Deep sequencing of miRNA (miRNA-seq) provided us with a unique opportunity to catalog the repertoire of miRNA expression and study miRNA dysregulation comprehensively. Of particular note, our analysis identified (in both the Discovery and Validation Cohorts) five known miRNA and one candidate novel miRNA (miR-28-5p, miR-324-5p, miR-214-5p, miR-339-3p, miR-5586-5p, NOVELM00203M) that are associated with patient survival independently of established indicators of outcome (cell-of-origin and International Prognostic Index scores). Our integrative analysis of miRNA-seq data with mRNA expression data from the same patients revealed that miRNAs that are upregulated in DLBCL appear to regulate genes involved in modulating the epigenome, and several of these are recurrently mutated in DLBCL as previously reported. It thus appears that dysregulation of the epigenome in DLBCL can be achieved through these different mechanisms. In addition, our comparison of DLBCL miRNA-seq expression profiles with those from 7,238 TCGA miRNA-seq libraries identified miRNAs (including three candidate novel miRNAs) that were more abundant in B-cell contexts, suggesting that these miRNAs may have B-cell specific functions in malignancy. Finally, this rich data set should prove valuable for researchers exploring DLBCL biology.

## Materials and methods

### Lymphoma patient samples (both Discovery and Validation Cohorts)

This project was approved by the University of British Columbia–BC Cancer Agency Research Ethics Board as part of a broad effort to increase understanding of the molecular biologic characteristics of lymphoid cancers (REB #H05-60103). Informed consent was obtained in accordance with the Declaration of Helsinki. Lymphoma samples were classified by an expert hematopathologist (RDG) according to the World Health Organization criteria of 2008.

### Patient sample acquisition (Discovery Cohort)

Benign specimens were purified CD77-positive centroblasts sorted from reactive tonsils using Miltenyi magnetic beads (Miltenyi Biotec, CA, USA). More details and the cell-of-origin subtype assignment (performed using RNA-seq expression values) are reported in Morin *et al.* [23]. RNA extraction was performed as reported in The Cancer Genome Atlas Research Network, 2013 [47].

### Patient sample acquisition (Validation Cohort)

These samples were obtained from FFPET blocks from which one to two 10 μm scrolls of each block were cut. Subsequently, total RNA, including miRNA, was extracted from FFPET tissues using AllPrep DNA/RNA FFPET (Qiagen) and High Pure (Roche) kits in a procedure developed by the TCGA project through the Biospecimen Core Resources at Nationwide Children’s Hospital and International Genomics Consortium (manuscript in preparation). The cell-of-origin subtype assignment was performed as reported in Scott *et al.* [48].

### Library Construction and Sequencing of miRNA-seq Illumina libraries

miRNA-seq library construction, sequencing, read alignment, and miRNA expression profiling was performed as reported in The Cancer Genome Atlas Research Network, 2013 [47]. Our threshold for calling expressed miRNAs (>10 RPM in >10% samples) was based on miRBase criteria [16] for high confidence miRNAs. The miRNA-seq bam files of DLBCL samples from both the discovery and validation cohorts and the centroblasts are uploaded on EGA (Study#: EGAS00001001025 and Data Set#(s): EGAD00001001073, EGAD00001001074, EGAD00001001075); web link: [49].

### Discovery of candidate novel miRNAs

Novel miRNA discovery was performed using mirDeep [50] in each of the 92 DLBCL miRNA-seq libraries. miRNA-seq reads were extracted from BAM files into a SAM format that was then analyzed using the mirDeep algorithm. As recommended by the authors of the software, only miRNA-seq reads >17 nucleotides in length were used for analysis. A list of all candidate novel miRNAs and their genomic coordinates was obtained from the results of each miRDeep run and then merged into a single file to eliminate duplicate entries. Merging was performed using a Perl script that considered overlapping genomic coordinates within +/− 2 bp. Each unique candidate novel miRNA was then given a name with the following format: ‘NOVEL[M/S]XXXXX’, where M and S indicated the mature or star strand respectively, and where the Xs represented a unique index number for each entry. In several instances, miRDeep had incorrectly identified other RNA species (that is, snoRNA, tRNA) as miRNA. These were identified by intersecting their coordinates with tracks supplied by UCSC [51] for these RNA species (using intersect of the bedtools package v2.16.2), and disregarded in subsequent analyses. NOVELM00113, NOVELM00156, NOVELM00203, NOVELM00289, and NOVELM00295 were retained for analysis, but we note that they also share sequence identity with mt-tRNA, RNU12, SOX2-OT, RNU4-82P, and RNA28S5, respectively, and thus may also be classified as other species of RNAs. The shortlisted genomic coordinates were then used as annotations in our miRNA profiling pipeline to assess the expression of the candidate novel miRNAs in all 92 DLBCL and 15 centroblast miRNA-seq libraries.

### Analysis of HITS-CLIP data

HITS-CLIP data from Haecker *et al.* [20] were obtained from the Sequence Read Archive (ID: SRR580359, SRR580360, SRR580361, SRR580362, SRR580363). The reads were aligned and processed for miRNA expression with the same protocols that were used for our miRNA-seq libraries.

### Quantitative RT-PCR for novel miRNA validation

To measure miRNA expression, leftover total RNA from tumor tissues utilized for miRNA sequencing were synthesized into cDNA using the Universal cDNA Synthesis Kit II (Exiqon, Denmark) and qPCR was performed using the ExiLENT SYBR Green master mix (Exiqon) following the manufacturer’s protocol. Reverse Transcription conditions used were: 42°C for 60 min, 95°C for 5 min, and stored at −20°C until ready for use. cDNA was diluted 1:80 prior to use for qPCR. qPCR conditions used were 40 cycles of 95°C for 10 s and 60°C for 1 min. All measurements were performed in triplicates. miRNA expression was normalized to endogenous RNU48 levels using the ∆∆Ct method.

### mRNA isoform-specific expression profiling with mRNA-seq

mRNA-seq sequence data were obtained from Morin *et al.* [23]. The mRNA-seq paired-end reads were aligned to RefSeq hg19 genome using TopHat v1.4.1 [52]. Alignments were then interrogated for isoform-specific expression profiles using Cufflinks v1.3.0 [52]. Only mRNA transcript isoforms that were expressed at 1 fragment per kilobase of million mapped reads (FPKM) in at least 10% of samples were considered for analysis.

### Differential expression analysis

Prior to differential expression analysis, miRNA expression profiles were quantile normalized using the R preprocessCore package. Evaluation of the differential expression of miRNA and mRNA was performed using the Wilcoxon ranked-sum test for each miRNA and mRNA. Significantly differentially expressed miRNA had Bejamini-Hochberg (BH) multiple test corrected *P* values (q-values) <0.05.

### Integrative miRNA:mRNA expression analysis

For the integrative miRNA:mRNA expression analysis we considered miRNAs and mRNA transcript isoforms that were expressed in >10% of DLBCL and centroblasts samples. A Spearman correlation coefficient (rho) score and *P* value was generated for each miRNA:mRNA pair. The *P* values were then multiple-test corrected for each miRNA with the BH algorithm. Significantly anti-correlated pairs were those that had Spearman correlation coefficient scores <0 and adjusted q-values <0.05. To account for correlations that might have been stochastic noise, the rho distribution was then divided in 40 bins and the counts for each bin compared with counts from a null distribution. miRNA:mRNA pairs in each bin were sorted by adjusted p-value, and only those that ranked above the threshold set by counts from bins derived from null distribution were considered for further analysis. The null distribution was derived by performing the Spearman correlations 100 times, each time randomizing the miRNA-seq library IDs.

Two algorithms were used for miRNA target prediction: TargetScan6.0 [53] and miRanda [54]. Target prediction was performed on all RefSeq hg19 mRNA transcript isoform sequences (including the 5’-UTR, CDS and 3’-UTR). While it is generally accepted that miRNAs target the 3’-UTR of mRNA transcripts, there are also reports of miRNA target sites in the CDS (that is, Forman *et al.* [55]; Duursma *et al.* [56]; Qin *et al.* [57]; Ott *et al.* [58]). In addition, the binding of miRNAs to binding sites within the 5’-UTR is as effective as binding to sites within the 3’-UTR [59]. Further, binding of miRNAs to CDS regions has been confirmed using large-scale high throughput approaches for isolating Argonuate-bound target sites. (Chi *et al.* [60]; Hafner *et al.* [61]). Thus, although evidence for binding sites in 5’-UTR and CDS regions is still accumulating, evidence for them exists in the literature and so we included them in our analysis along with those within the 3’-UTR. Although we required that candidate binding sites be identified using both TargetScan6.0 [53] and miRanda [54], it is possible that certain predictions represent false positives.

miRNA sequences and input data for annotated miRNAs was obtained from TargetScan and miRanda, respectively, while candidate novel miRNA sequences were obtained from miRNA-seq consensus sequences. miRNA:mRNA pairs were considered to have a miRNA-mediated repression interaction if they had anti-correlated expression profiles and where the miRNA had a predicted binding site (determined by both algorithms) on the mRNA.

### Gene Ontology (GO) term enrichment analysis

GO term enrichment analysis was performed using the MGSA (v 1.10.0) R package [62]. The lists of predicted target genes (obtained from the integrative expression analysis) for each miRNA were assessed separately for enriched GO Bioprocess terms. Significant terms were those with standard error measurements <0.05 and estimates >0.2. To assess whether groups of miRNAs (that is, where a group might consist of miRNAs that are upregulated in DLBCL), together enriched particular GO Terms more so than by random chance, a Fisher’s Exact test was performed for each enriched term. The numbers of miRNAs in the category and out of the category that enriched the GO term were compared. The Fisher’s Exact Test *P* values were then multiple-test corrected with the BH algorithm, where significant enrichments by a category were those with q-values <0.05.

### Cell culture

HEK-293 cells were maintained in Dulbecco’s Modified Eagle Medium (DMEM; Life Technologies, Burlington ON) supplemented with 10% (v/v) fetal bovine serum (FBS; Life Technologies) in a 37°C incubator with 5% CO2, humidified atmosphere.

### Plasmid constructs

The *MLL2/KMT2D* or *EP300* genomic or mismatched sequences corresponding to the predicted miR-10393-3p binding sites were synthesized (IDT Technologies, Coralville, IA, USA) and cloned into the *XhoI/NotI* restriction sites of the psiCHECK2 vector (Promega, Madison, WI, USA) directly downstream of the Renilla luciferase reporter gene and verified by DNA sequence analysis. The mismatched sequences were designed to be exactly complementary to the seven nucleotide seed regions of each of the predicted miR-10393-3p binding sites to *MLL2/KMT2D* or *EP300*.

### MicroRNA mimics

MicroRNA expression was increased using MIRIDIAN microRNA mimics (ThermoScientific, Waltham MA) directed against miR-10393-3p (M10393; 5’-UUGGUCAGAUUUGAACUCUUCA-3’) and negative control #2 (NC2; non targeting control against *C. elegans* cel-miR-239b). Mimics were resuspended in nuclease-free water at a stock concentration of 100 μM.

### Dual-Luciferase reporter assays

HEK-293 cells were seeded onto 24-well plates the day before transfection. Perfect binding or mismatched reporter constructs were co-transfected with miR-10393-3p mimics or NC2 control mimics using TurboFect Transfection Reagent (ThermoScientific) in OPTI-MEM (Life Technologies) without FBS. Six hours following transfection, the media was changed to DMEM supplemented with 10% FBS. Cells were reseeded the following day into 96-well plates and 48h following transfection, cells were lysed and luciferase activities were assayed using the Dual-Glo Luciferase Reporter Assay System (Promega). The Renilla to Firefly luciferase ratios were calculated for each well to account for transfection efficiencies. These experiments were performed in quadruplicates and were shown as means ± SEM. Statistical comparisons were performed using unpaired two-tailed T-tests with Bonferroni multiple-test correction, where significant differences were those with adjusted *P* value <0.05.

### Survival analysis

Progression-free survival (PFS; event = progression of disease or death from any cause) and overall survival (OS; event = death from any cause) were estimated. For each miRNA, we used X-tile cohort separation [24] to categorize patients into low and high expression groups, and then performed log-rank tests based on these derived groups. For the multivariate analysis for each miRNA, we considered the aforementioned low and high expression groups along with COO and IPI status using the Cox proportional hazards (Cox PH) method. All calculations were performed using the Survival R package [63]. Survival analyses were performed as above for both the discovery and validation cohorts. Significant associations with survival were those with *P* value <0.05. In addition, *P* values obtained from the log-rank tests in the discovery cohort were subjected to multiple-test correction using the BH algorithm, and significant associations for that analysis were those with corrected *P* values (q-values) <0.05.

### NMF clustering of miRNA-seq expression

miRNAs that were expressed at levels >10 RPM in at least 10% of the 92 DLBCL and 15 centroblast samples were included in the NMF clustering analysis. Because we were interested in assessing associations with outcome between groups of patients, we only considered the data from the 83 patients that were uniformly treated with R-CHOP for this clustering analysis. We generated unsupervised consensus clustering results as described in The Cancer Genome Atlas Research Network, 2013 [47]. We used the default Brunet algorithm and 100 iterations for the rank survey and clustering runs. A preferred cluster result was selected by considering the profiles of the cophenetic scores of the consensus membership matrix for clustering solutions having between two and eight clusters. We chose the 2-group (k = 2) solution as it had the second highest cophenetic score and produced a visually clean consensus matrix when compared with the other solutions (Additional file 9: Figure S4). Since some of the k = 3 to 8 solutions have relatively high cophenetic scores, there is likely heterogeneity within ‘cluster 2’ of the k = 2 solution. However, we chose to present the k = 2 solution because the focus of our analysis was on the characterization of ‘cluster 1’, the cluster that does not lose its integrity as we increase the number of clusters. That is, in the k = 8 solution, ‘cluster 1’ (from the k = 2 solution) still appears as a distinct cluster of patients with poor outcome that is characterized by reduced expression of miR-21 and abundant expression of miR-148a.

## Additional files

Additional file 1: Table S1. Clinical characteristics of the 83 patients with *de novo* DLBCL (Discovery Cohort). Additional file 2: Table S2. Detailed clinical characteristics of each patient in the Discovery and Validation Cohorts. Additional file 3: Table S3. miRNA-seq alignment statistics for each DLBCL and Centroblast library. Additional file 4: Table S4. Genomic coordinates of identified candidate novel miRNAs. Additional file 5: Table S5. miRNA:mRNA interactions displaying anti-correlated expression profiles, filtered by TargetScan and miRanda predictions. Additional file 6: Table S6. Novel miRNA validation results. HITS-CLIP expression and validation cohort expression for the 30 candidate novel miRNAs. Additional file 7: Table S7. Tables of miRNA sequencing vs. RT-qPCR results for validation of selected candidate novel miRNAs. Additional file 8: Table S8. Differentially expressed miRNAs when comparing (1) B-cell data sets to all TCGA cancer data sets; (2) DLBCL to Centroblast samples; (3) ABC-DLBCL to GCB-DLBCL samples. Additional file 9: Figure S1. miR-142 expression in DLBCL, centroblasts, and other cancers. **Figure S2.** Pipeline for discovering putative miRNA:mRNA interactions acting in DLBCL. **Figure S3.** Kaplan-Meier (KM) Curves Illustrating DLBCL Patient Survival. **Figure S4.** Non-Negative Matrix Factorization (NMF) Solutions. **Figure S5.** miR-148a and miR-21 expression levels are associated with survival. **Figure S6.** Heatmap Comparing Matched Discovery Cohort (fresh frozen (FF)) and Validation Cohort (formalin-fixed, paraffin-embedded (FFPE)) samples for 28 cases. **Figure S7.** Kaplan-Meier curves and strip charts of expression levels for the six miRNAs that were found to be associated with OS and PFS, independently of COO and IPI in both the Discovery and Validation Cohorts. Additional file 10: Table S9. GO Terms enriched by targets of various categories of miRNAs. Additional file 11: Table S10. miRNAs associated with DLBCL patient overall survival (OS) and progression-free survival (PFS) in Discovery and Validation Cohorts. Additional file 12: Table S11. Clinical characteristics of the 112 patients with *de novo* DLBCL (Validation Cohort). Additional file 13: Table S12. miRNA expression levels in DLBCL cell lines from Jima *et al.* and in our fresh frozen DLBCL samples (Discovery Cohort).

## Abbreviations

ABC: Activated B-cell-like
ALL: Acute lymphoblastic leukemia
BH: Benjamini-Hochberg
CDS: Coding region/sequence
CHOP: Combination of cyclophosphamide, doxorubicin, vincristine and prednisone chemotherapy
COO: Cell-of-origin
DLBCL: Diffuse large B-cell lymphoma
DNA: Deoxyribonucleic acid
FFPET: Formalin-fixed, paraffin-embedded tissue
FPKM: Fragment per kilobase of million mapped reads
GCB: Germinal centre B-cell-like
GO: Gene Ontology
IPI: International Prognostic Index
LDH: Lactate dehydrogenase
miRISC: RNA-induced silencing complex
miRNAs: microRNAs
mRNA: Messenger RNA
NHL: Non-Hodgkin lymphoma
NMF: Non-negative matrix factorization
OS: Overall survival
PFS: Progression-free survival
qPCR: Quantitative polymerase chain reaction
R-CHOP: Combination of cyclophosphamide, doxorubicin, vincristine and prednisone chemotherapy with rituximab
RNA: Ribonucleic acid
RPM: Reads per million mapped reads
rRNA: Ribosomal RNA
scRNA: Small cytoplasmic RNA
snoRNA: Small nucleolar RNA
snRNA: Small nuclear RNA
TCGA: The Cancer Genome Atlas
tRNA: Transfer RNA
UCSC: University of California, Santa Cruz
UTR: Untranslated region
