# Supplementary material for: Comprehensive miRNA sequence analysis reveals survival differences in diffuse large B-cell lymphoma patients
Source: Genome Biol. 2015 Jan 29;16(1):18. doi: 10.1186/s13059-014-0568-y (PMC4308918; doi:10.1186/s13059-014-0568-y)
Supplement: Additional file 1: Table S1. — Clinical characteristics of the 83 patients with de novo DLBCL (Discovery Cohort). [file 13059_2014_568_MOESM1_ESM.docx]

**Supplementary Table S1. Clinical characteristics of the 83 patients with *de novo* DLBCL (Discovery Cohort).**

| **Demographic or clinical characteristic** | **All patients**  **(24 patients)** |
| --- | --- |
| **Male (%)** | 61 |
| **Age (years) (median (range))** | 66 (16 to 92) |
| **Stage (n (%))** |  |
| I/II | 39 (50) |
| III/IV | 40 (48) |
| NA | 4 |
| **Lactate dehydrogenase > ULN (n (%))** |  |
| No | 31 (37) |
| Yes | 35 (42) |
| NA | 17 |
| **ECOG performance status (n (%))** |  |
| 0 to 1 | 57 (68) |
| At least 2 | 22 (27) |
| NA | 4 |
| **Extranodal sites (n (%))** |  |
| 0 to 1 | 70 (84) |
| Greater than 1 | 9 (11) |
| NA | 4 |
| **Revised International Prognostic Index^a^ (n) (%)** |  |
| Very Good and Good (0 to 2) | 52 (63) |
| Poor (3 to 5) | 22 (27) |
| NA | 9 |
| **Cell-of-origin^b^ (n (%))** |  |
| GCB | 41 (49) |
| ABC | 29 (35) |
| Unclassified | 8 (10) |
| NA | 5 |
| ***BCL2* FISH breakapart^c^ (n (%))** |  |
| Positive | 20 (24) |
| Negative | 39 (47) |
| NA | 24 |
| ***BCL6* FISH breakapart^c^ (n (%))** |  |
| Positive | 10 (12) |
| Negative | 48 (58) |
| NA | 25 |
| ***MYC* FISH breakapart^c^ (n (%))** |  |
| Positive | 6 (7) |
| Negative | 52 (63) |
| NA | 25 |
| **B symptoms (n (%))** |  |
| Absent | 50 (60) |
| Present | 25 (30) |
| NA | 8 |

ECOG: Eastern Cooperative Oncology Group; GCB: germinal center B-cell like; NA: not available; ULN: upper limit of normal.

^a^The Revised International Prognostic Indicator (R-IPI) score ranges from 0 to 5, with higher scores indicating increased risk [1,2].

^b^Cell-of-origin (COO) was determined by RNA-seq gene expression profiling using the Wright *et al.* [3] classifier.

^c^The presence of translocations was determined using commercial dual color ‘break-apart’ probes from Abbott Molecular (Abbot Park, IL, US) on tissue microarray using the method described in Chin *et al.* [4].

**References**

1. The International Non-Hodgkin’s Lymphoma Prognostic Factors Project. A predictive model for aggressive non-Hodgkin‘s lymphoma. The International Non-Hodgkin’s Lymphoma Prognostic Factors Project. N Engl J Med. 1993;329:987–94.

2. Sehn LH Berry B, Chhanabhai M, Fitzgerald C, Gill K, Hoskins P, et al. The revised International Prognostic Index (R-IPI) is a better predictor of outcome than the standard IPI for patients with diffuse large B-cell lymphoma treated with R-CHOP. Blood. 2007;109:1857–.

3. Wright G. A gene expression-based method to diagnose clinically distinct subgroups of diffuse large B cell lymphoma. Proc Natl Acad Sci U S A. 2003;100:9991–6.

4. Chin SF, Daigo Y, Huang HE, Iyer NG, Callagy G, Kranjac T, et al. A simple and reliable pretreatment protocol facilitates fluorescent in situ hybridisation on tissue microarrays of paraffin wax embedded tumour samples. Mol Pathol. 2003;56:275–9.
