# Supplementary material for: Comprehensive miRNA sequence analysis reveals survival differences in diffuse large B-cell lymphoma patients
Source: Genome Biol. 2015 Jan 29;16(1):18. doi: 10.1186/s13059-014-0568-y (PMC4308918; doi:10.1186/s13059-014-0568-y)
Supplement: Additional file 9: Figure S1. — miR-142 expression in DLBCL, centroblasts, and other cancers. Figure S2. Pipeline for discovering putative miRNA:mRNA interactions acting in DLBCL. Figure S3. Kaplan-Meier (KM) Curves Illustrating DLBCL Patient Survival. Figure S4. Non-Negative Matrix Factorization (NMF) Solutions. Figure S5. miR-148a and miR-21 expression levels are associated with survival. Figure S6. Heatmap Comparing Matched Discovery Cohort (fresh frozen (FF)) and Validation Cohort (formalin-fixed, paraffin-embedded (FFPE)) samples for 28 cases. Figure S7. Kaplan-Meier curves and strip charts of expression levels for the six miRNAs that were found to be associated with OS and PFS, independently of COO and IPI in both the Discovery and Validation Cohorts. [file 13059_2014_568_MOESM9_ESM.pdf]

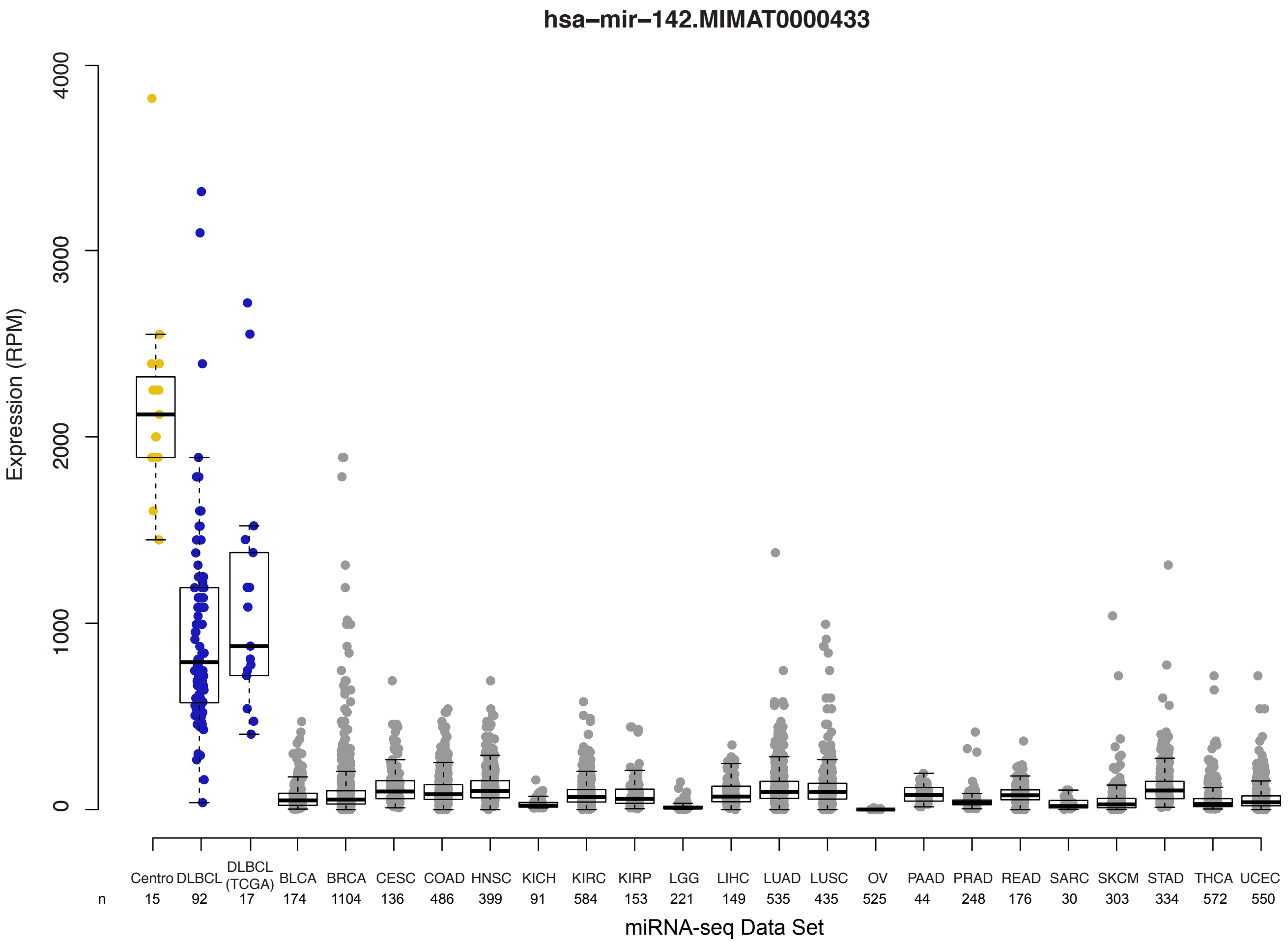


**Supplementary Figure S1 – miR-142 expression in DLBCL, centroblasts, and other cancers.** BLCA (Bladder Urothelial Carcinoma); BRCA (Breast invasive carcinoma) CESC (Cervical squamous cell carcinoma and endocervical adenocarcinoma); COAD (Colon adenocarcinoma); HNSC (Head and Neck squamous cell carcinoma); KICH (Kidney Chromophobe); KIRC (Kidney renal clear cell carcinoma); KIRP (Kidney renal papillary cell carcinoma); LGG (Brain Lower Grade Glioma); LIHC (Liver hepatocellular carcinoma) LUAD (Lung adenocarcinoma); LUSC (Lung squamous cell carcinoma); OV (Ovarian serous cystadenocarcinoma); PAAD (Pancreatic adenocarcinoma); PRAD (Prostate adenocarcinoma); READ (Rectum adenocarcinoma); SARC (Sarcoma); SKCM (Skin Cutaneous Melanoma ); STAD (Stomach adenocarcinoma); THCA (Thyroid carcinoma); UCEC (Uterine Corpus Endometrial Carcinoma); Blue: DLBCL; Orange: Centroblast.

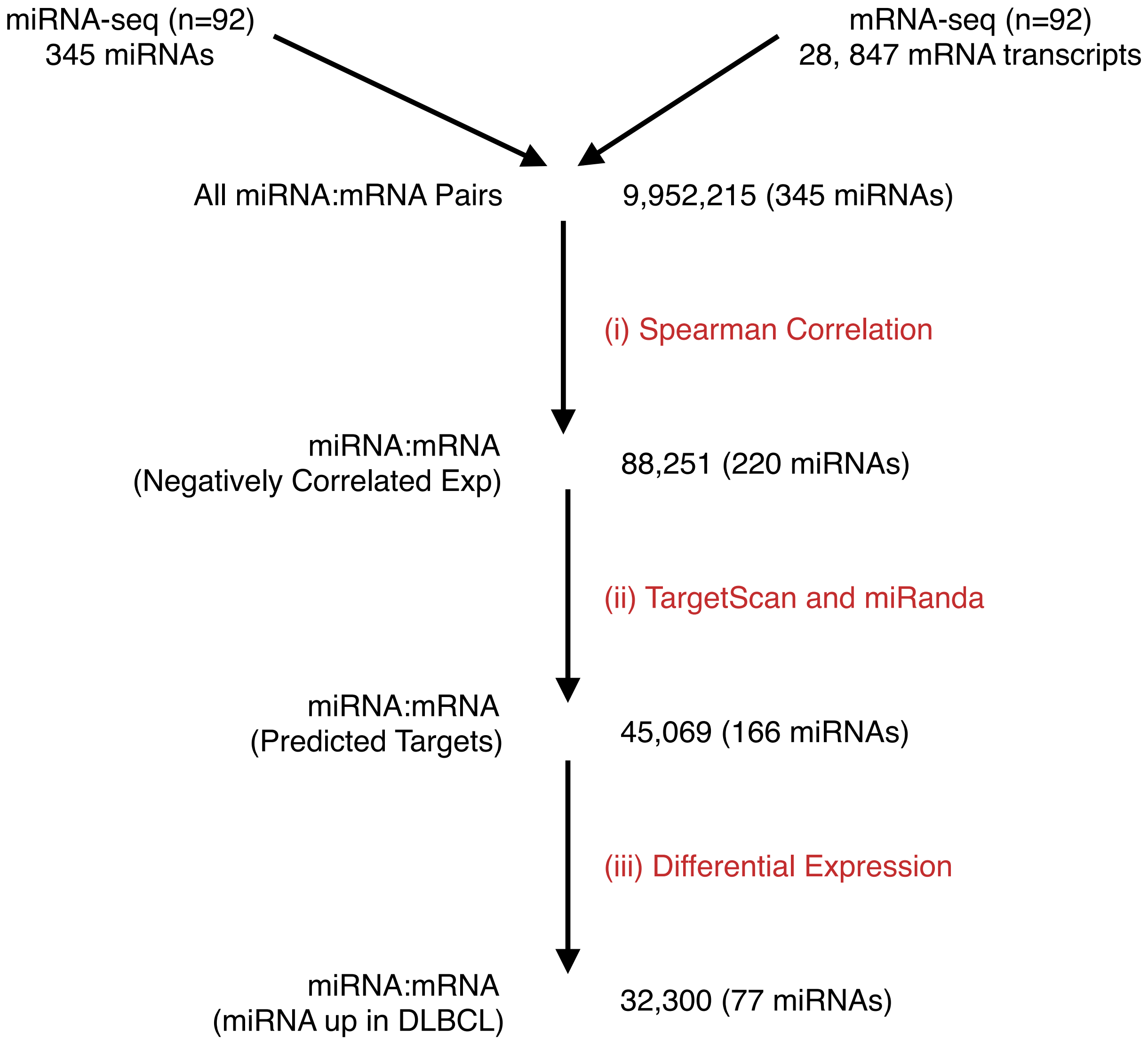


**Supplementary Figure S2 – Pipeline for discovering putative miRNA:mRNA interactions acting in DLBCL.** For this analysis we utilized miRNA-seq and mRNA-seq expression profiles from 92 DLBCL patients. (i) To identify miRNA:mRNA pairs with anti-correlated expression profiles we performed Spearman correlations on each miRNA:mRNA pair, and shortlisted the interactions with Spearman correlation coefficients <0 (BH q-value<0.05). (ii) We then filtered these interactions through TargetScan and miRanda predictions to obtain interactions where the mRNA had the binding site for the miRNA. (iii) Finally we considered only interactions involving a miRNA that was increased in abundance in DLBCL when compared with benign centroblasts, where the up-regulated miRNAs were determined by differentially expression analysis (Wilcoxon test, BH q-value <0.05).

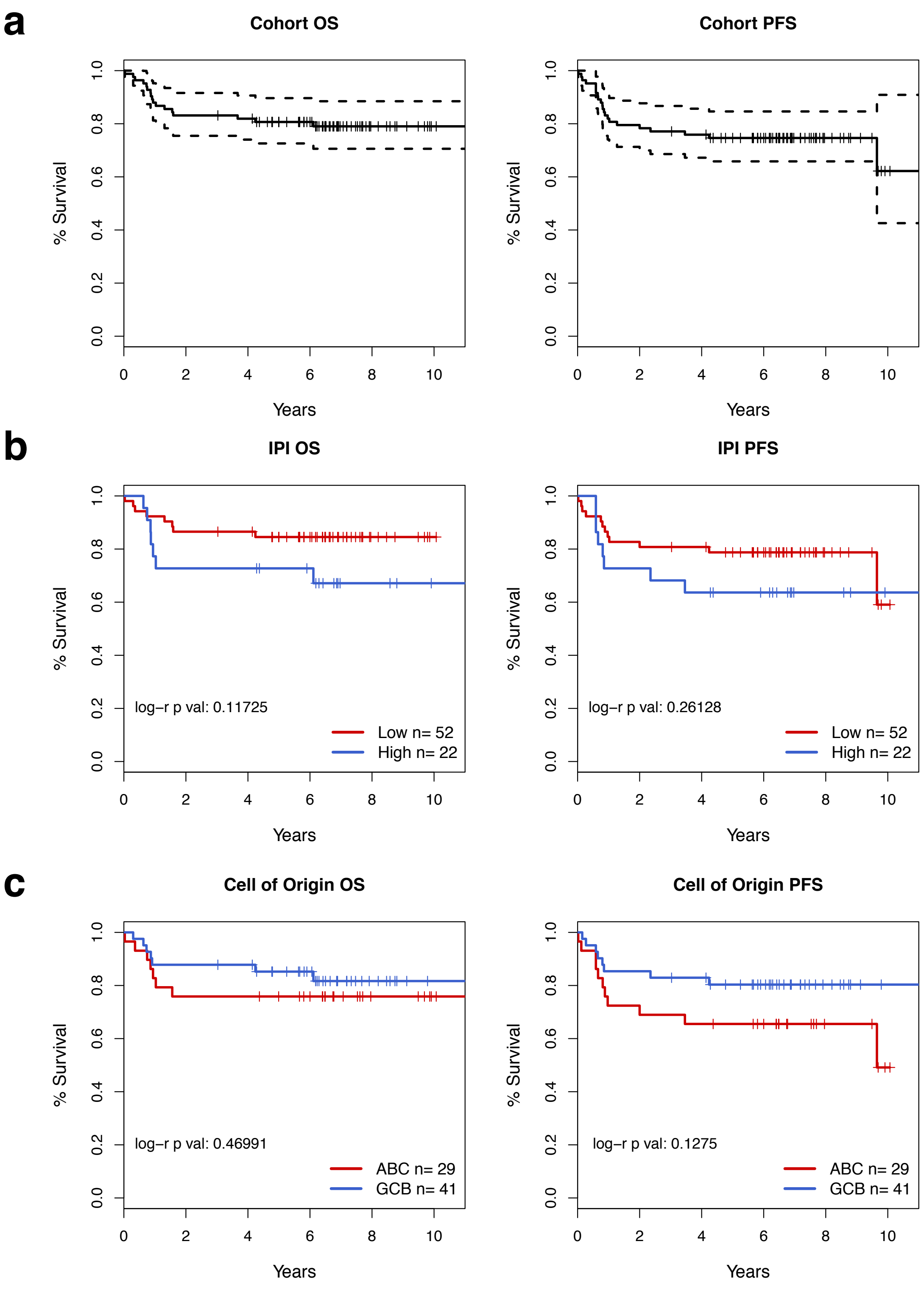


**Supplementary Figure S3 – Kaplan-Meier (KM) Curves Illustrating DLBCL Patient Survival** - We had 83 R-CHOP treated patients for which we had overall survival (OS) and progression free survival (PFS) data. A) KM curves for the entire cohort. The dotted line above and below represent that confidence interval. B) KM curves of patients stratified by International Prognostic Scores (IPI) (Low: 0-2; High 3-5). C) KM curves of patients stratified by tumor cell-of-origin (COO): ABC (Activated B-cell-like); GCB (Germinal Center B-cell-like). COO status was derived using a gene expression signature from mRNA-seq data**.**

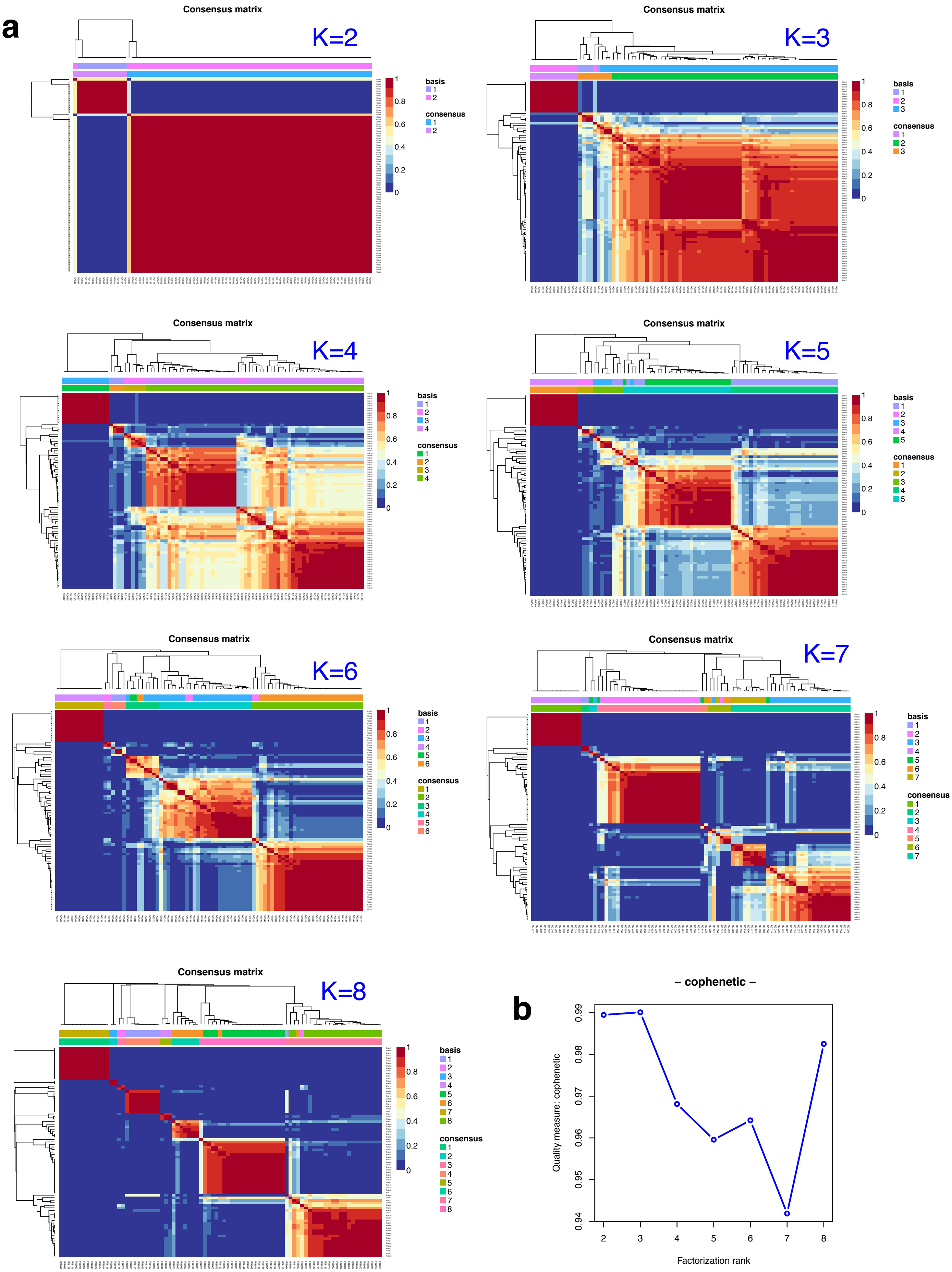

a
Consensus matrix
K=2
K=3
K=4
K=5
K=6
K=7
K=8
basis
consensus
b
– cophenetic –
Quality measure: cophenetic
Factorization rank

**Supplementary Figure S4 – Non-Negative Matrix Factorization (NMF) Solutions** - A) Consensus matrices from different NMF solutions for K=2:8. B) Cophenetic scores from different solutions for K=2:8. We selected the K=2 solution for further analysis as the consensus matrix appeared to be the cleanest, and had the highest cophenetic coefficient.

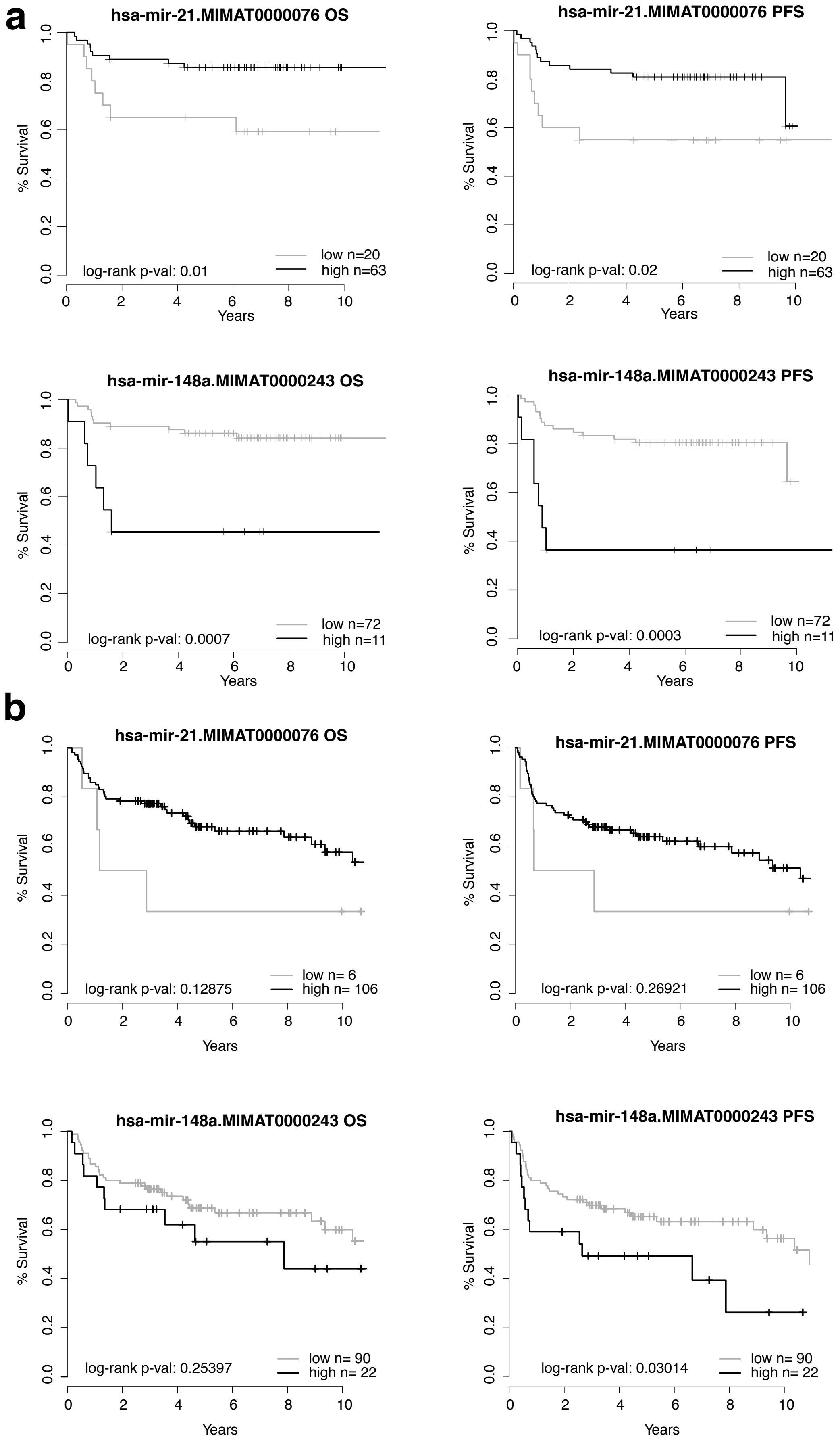


**Supplementary Figure S5 – miR-148a and miR-21 expression levels are associated with survival.** Kaplan-Meier plots show that miR-148a is positively correlated with poor outcome, and that miR-21 is negatively correlated with poor outcome in both the discovery cohort (a) and validation cohort (b).

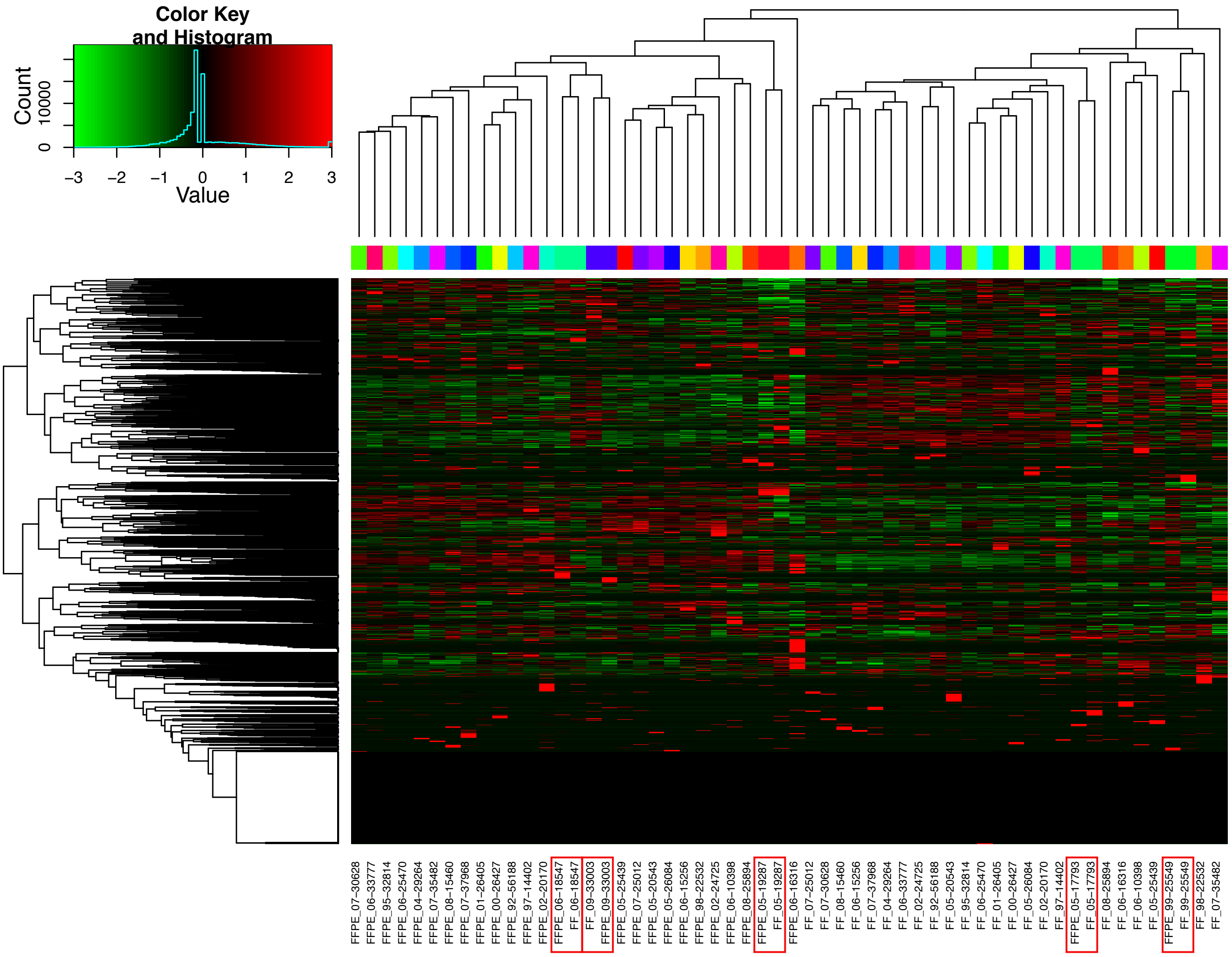


**Supplementary Figure S6 – Heatmap Comparing Matched Discovery Cohort (fresh frozen – FF) and Validation Cohort (formalin-fixed, paraffin-embedded - FFPE) Samples for 28 Cases.** Unsupervised clustering of miRNA expression profiles from matched FF and FFPE samples of 28 cases, from the discovery and additional cohorts respectively, indicates that FF and FFPE samples have distinct expression profiles. With the exception of 5 cases which have their FF and FFPE samples clustering closest to one another (indicated in red boxes around sample labels), all FF and FFPE samples fall within the 2 distinct clusters representing FF and FFPE samples. Expression profiles from FF and FFPE samples were normalized by the total number of mapped reads in each library and then quantile normalization was performed across all 56 samples. The value plotted in the heatmap is a z-score of the expression of each miRNA in each sample.

# miR-28-5p

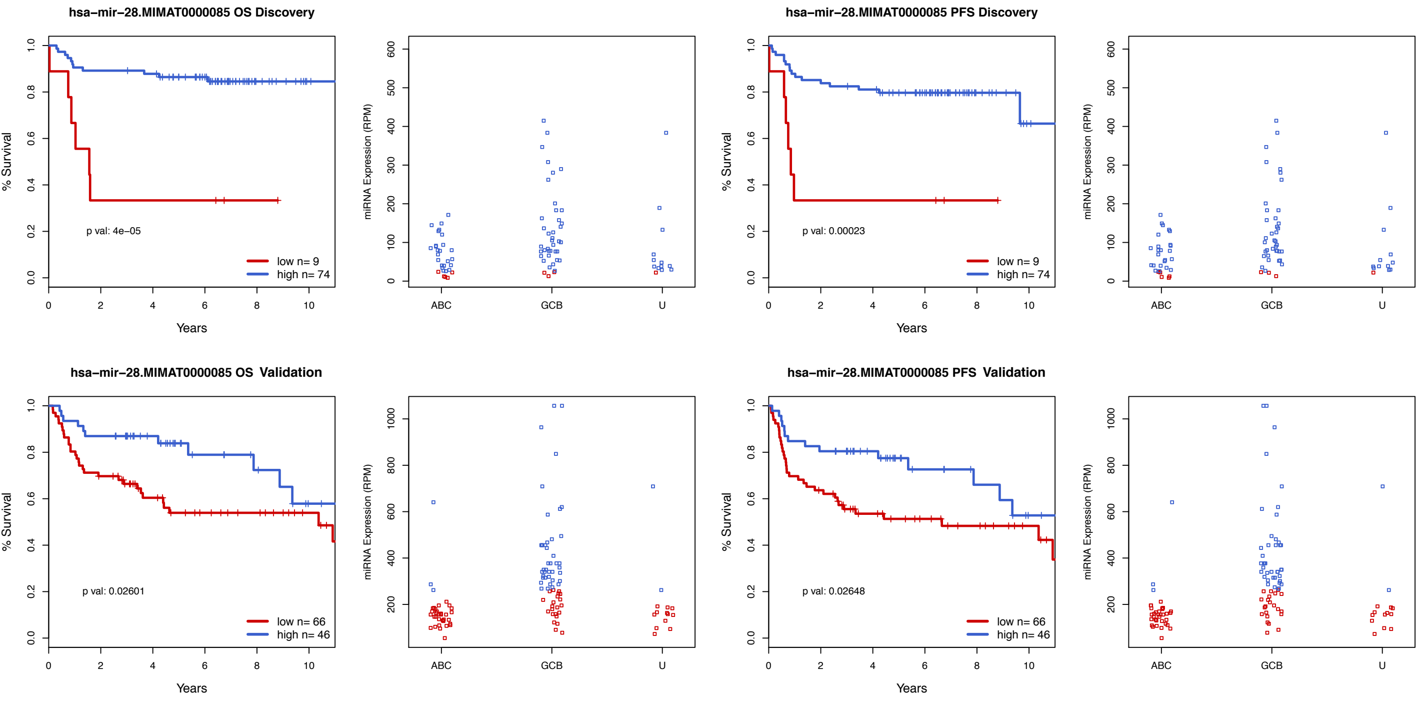


# miR-324-5p

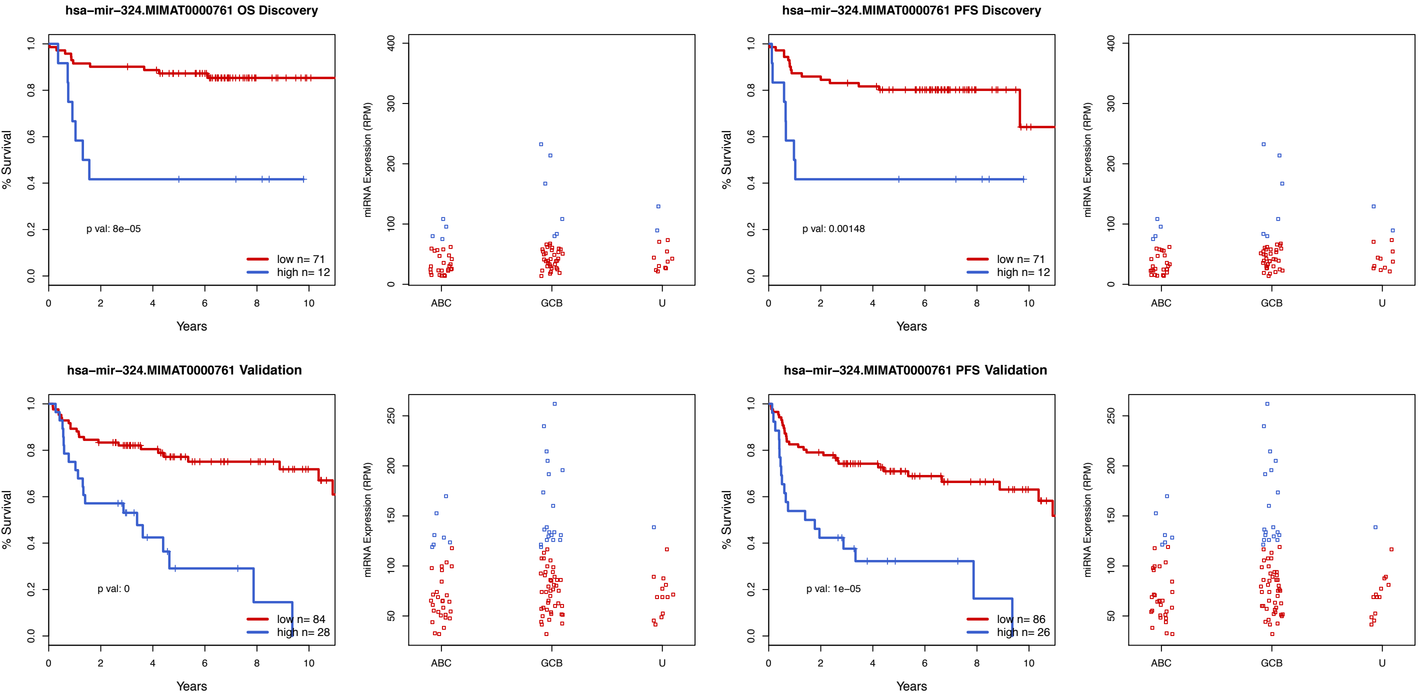


**Supplementary Figure S7**

# miR-214-5p

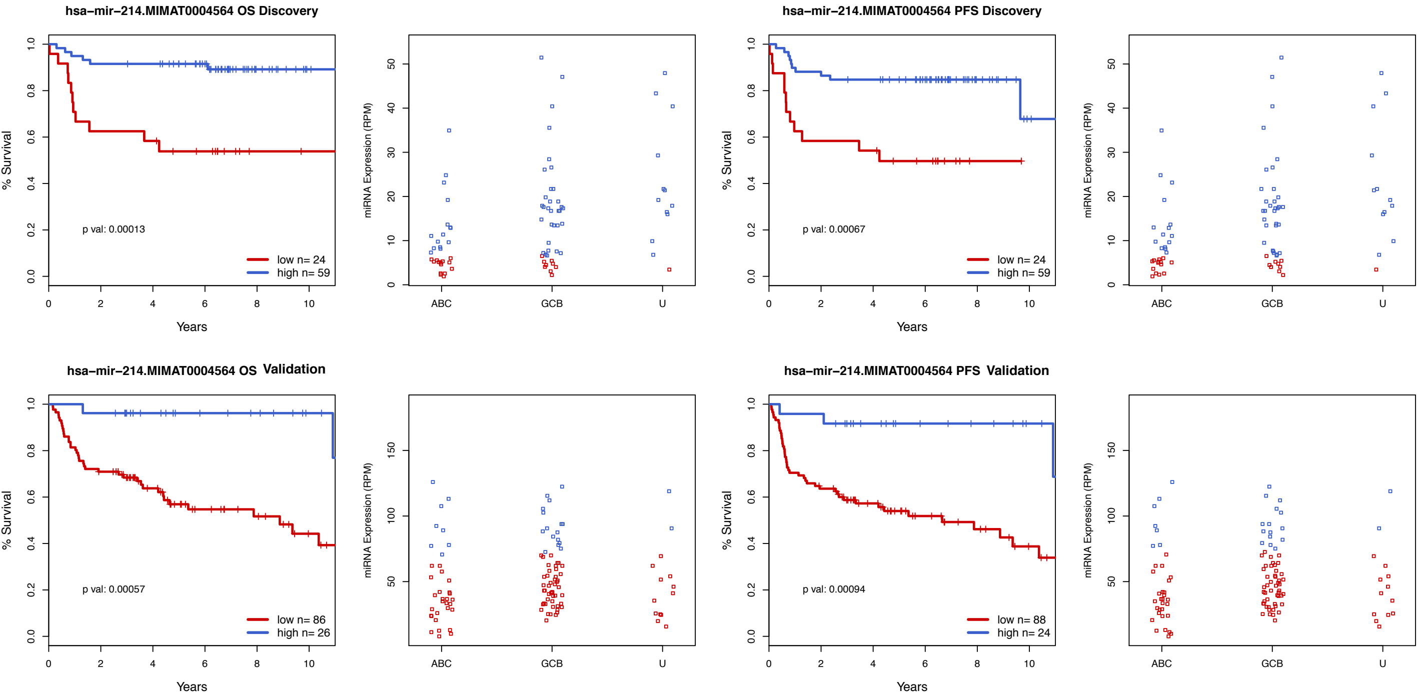


# NOVELM00203M

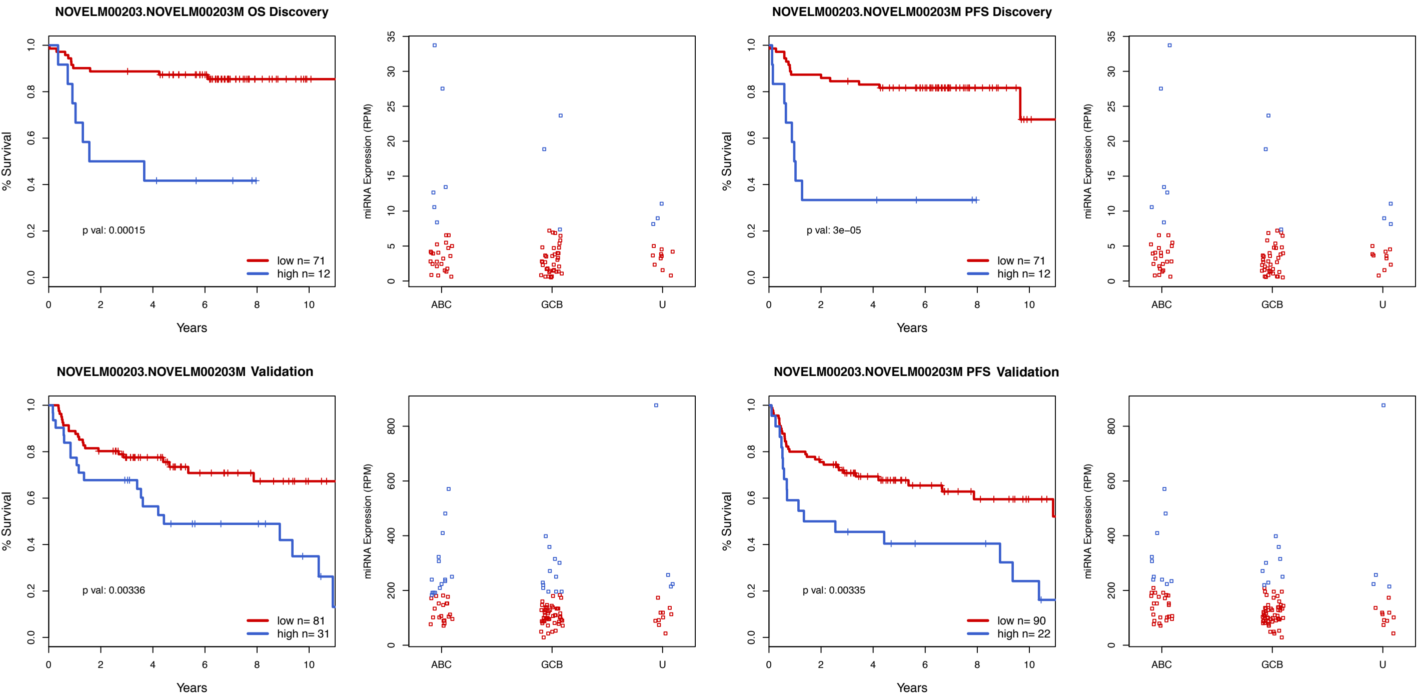


**Supplementary Figure S7 (cont')**

# miR-339-3p

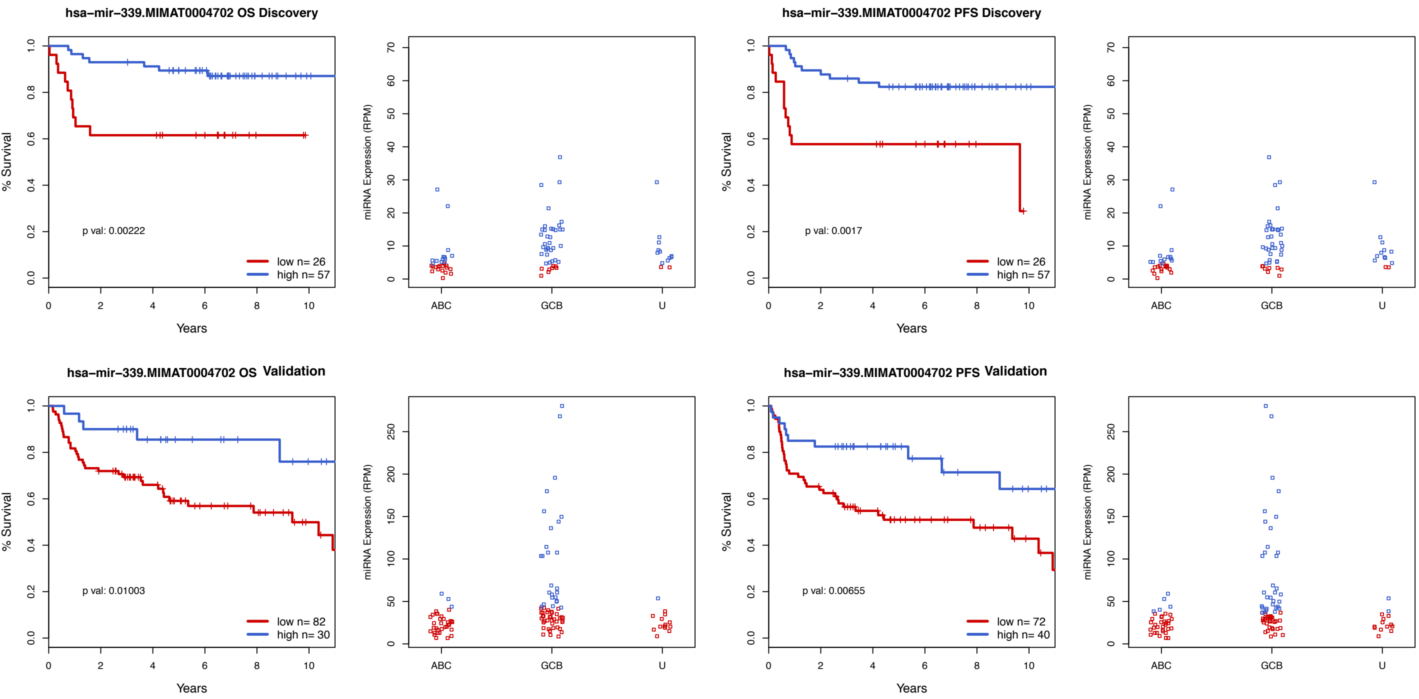


# miR-5586-5p

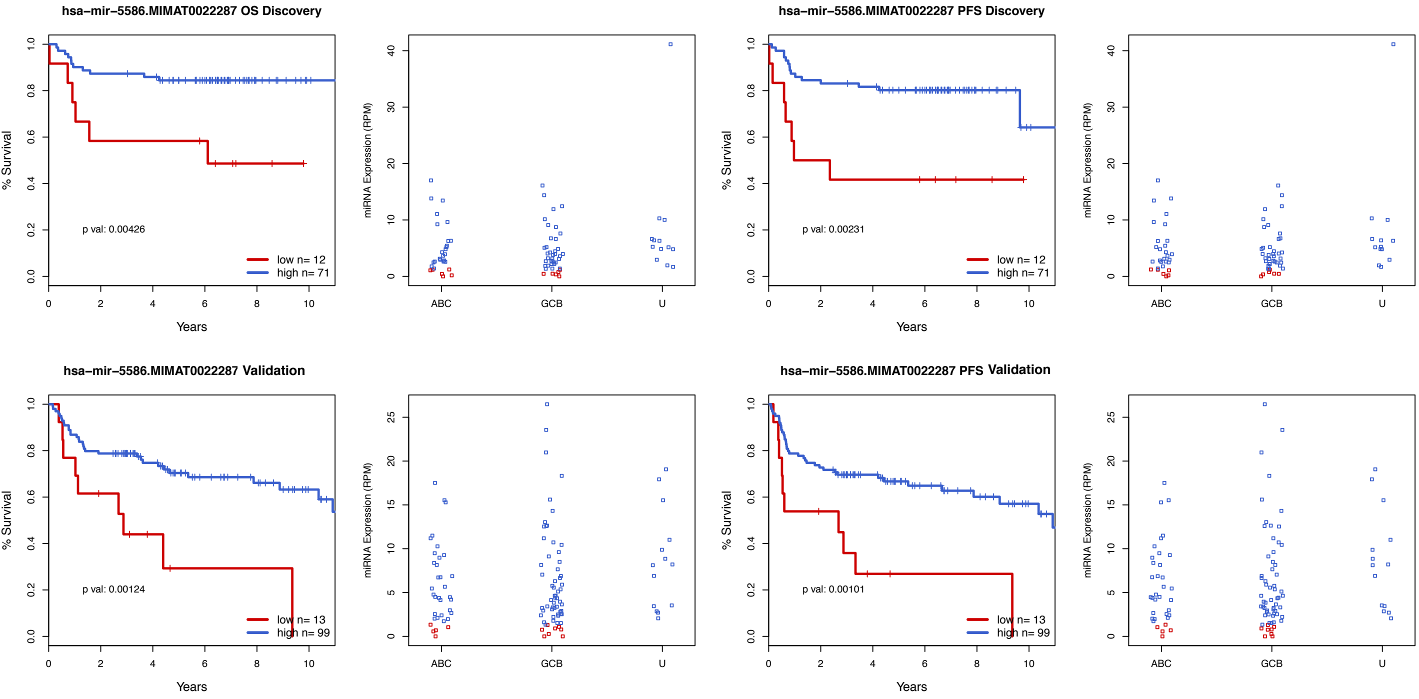


**Supplementary Figure S7 (cont') –** Kaplan-Meier curves and strip charts of expression levels for the 6 miRNAs that were found to be associated with OS and PFS, independently of COO and IPI in both the Discovery and Validation cohorts.
