# Supplementary material for: Comprehensive miRNA sequence analysis reveals survival differences in diffuse large B-cell lymphoma patients
Source: Genome Biol. 2015 Jan 29;16(1):18. doi: 10.1186/s13059-014-0568-y (PMC4308918; doi:10.1186/s13059-014-0568-y)
Supplement: Additional file 12: Table S11. — Clinical characteristics of the 112 patients with de novo DLBCL (Validation Cohort). [file 13059_2014_568_MOESM12_ESM.docx]

**Supplementary Table S11. Clinical characteristics of the 112 patients with *de novo* DLBCL (Validation Cohort).**

| **Demographic or clinical characteristic** | **All patients**  **(24 patients)** |
| --- | --- |
| **Male (%)** | 60 |
| **Age (years) (median (range))** | 65 (20 to 89) |
| **Stage (n (%))** |  |
| I/II | 51 (86) |
| III/IV | 59 (53) |
| NA | 2 |
| **Lactate dehydrogenase (median (range))** | 1.1 (0.57 to 17.1) |
| **ECOG performance status (n (%))** |  |
| 0 to 1 | 75 (67) |
| At least 2 | 35 (31) |
| NA | 2 |
| **Extranodal sites (n (%))** |  |
| 0 to 1 | 50 (44) |
| Greater than 1 | 19 (17) |
| NA | 43 |
| **Revised International Prognostic Index^a^ (n (%))** |  |
| Very Good and Good (0 to 2) | 69 (62) |
| Poor (3 to 5) | 37 (33) |
| NA | 6 |
| **Cell-of-Origin^b^ (n (%))** |  |
| GCB | 63 (56) |
| ABC | 35 (31) |
| Unclassified | 14 (13) |
| ***BCL2* FISH breakapart^c^ (n (%))** |  |
| Positive | 36 (32) |
| Negative | 69 (61) |
| NA | 7 |
| ***BCL6* FISH breakapart^c^ (n (%))** |  |
| Positive | 25 (22) |
| Negative | 80 (71) |
| NA | 7 |
| ***MYC* FISH breakapart^c^ (n (%))** |  |
| Positive | 86 (77) |
| Negative | 15 (13) |
| NA | 11 |

ECOG: Eastern Cooperative Oncology Group; GCB: germinal center B-cell like; NA: not available.

^a^The Revised International Prognostic Indicator (R-IPI) score ranges from 0 to 5, with higher scores indicating increased risk [1,2].

^b^Cell-of-origin (COO) was determined by the Lymph2CX [3] classifier.

^c^The presence of translocations was determined using commercial dual color ‘break-apart’ probes from Abbott Molecular (Abbot Park, IL, US) on tissue microarray using the method described in Chin *et al.* [4].

**References**

1. The International Non-Hodgkin’s Lymphoma Prognostic Factors Project. A predictive model for aggressive non-Hodgkin‘s lymphoma. The International Non-Hodgkin’s Lymphoma Prognostic Factors Project. N Engl J Med. 1993;329:987–94.

2. Sehn LH Berry B, Chhanabhai M, Fitzgerald C, Gill K, Hoskins P, et al. The revised International Prognostic Index (R-IPI) is a better predictor of outcome than the standard IPI for patients with diffuse large B-cell lymphoma treated with R-CHOP. Blood. 2007;109:1857–.

3. Wright G. A gene expression-based method to diagnose clinically distinct subgroups of diffuse large B cell lymphoma. Proc Natl Acad Sci U S A. 2003;100:9991–6.

4. Chin SF, Daigo Y, Huang HE, Iyer NG, Callagy G, Kranjac T, et al. A simple and reliable pretreatment protocol facilitates fluorescent in situ hybridisation on tissue microarrays of paraffin wax embedded tumour samples. Mol Pathol. 2003;56:275–9.
